# The missing link: Piccolino is essential for tethering synaptic vesicles to rod photoreceptor ribbons

**DOI:** 10.1083/jcb.202509110

**Published:** 2026-07-23

**Authors:** Kaspar Gierke, Michalina Gadomska, Julia Breuer, Julius N. Bahr, Tanja M. Müller, Hanna Ehnis, Sina Zobel, Nancy Mejia Villagran, Sonja A. Kirsch, Alexandra Skrzypek, Renato Frischknecht, Anna Fejtová, Rainer A. Böckmann, Carolin Wichmann, Hanna Regus-Leidig, Johann Helmut Brandstätter

**Affiliations:** 1 Department of Biology, Animal Physiology/Neurobiology, Friedrich-Alexander-Universität Erlangen-Nürnberg, Erlangen, Germany; 2 Center for Biostructural Imaging of Neurodegeneration (BIN), University Medical Center Göttingen, Göttingen, Germany; 3 Molecular Architecture of Synapses Group, Institute for Auditory Neuroscience and InnerEarLab, University Medical Center Göttingen, Göttingen, Germany; 4 Göttingen Graduate School for Neuroscience, Biophysics and Molecular Biosciences, University of Göttingen, Göttingen, Germany; 5 Department of Biology, Computational Biology, Friedrich-Alexander-Universität Erlangen-Nürnberg, Erlangen, Germany; 6 Erlangen National High-Performance Computing Center (NHR@FAU), Erlangen, Germany; 7 FAU Profile Center Immunomedicine (FAU I-MED), Erlangen, Germany; 8 Department of Psychiatry and Psychotherapy, Universitätsklinikum Erlangen, Friedrich-Alexander-Universität Erlangen-Nürnberg, Erlangen, Germany

## Abstract

Retinal photoreceptors transmit light signals to their postsynaptic neurons with high precision, speed and without fatigue. This high-throughput neurotransmission relies on a sophisticated molecular machinery centered on a presynaptic organelle, the synaptic ribbon (SR). A hallmark of SRs is the recruitment of synaptic vesicles (SVs) from the cytoplasmic SV pool via “tethering”. However, the identity of the tether and the mechanism underlying SV tethering are unknown. Here, we show that cell-specific deletion of the SR-associated protein Piccolino from rod photoreceptors disrupts SR morphology and ablates SV tethering. Nanoscale epitope mapping suggests that Piccolino acts as an SV tether by extending its N terminus away from the SR into the SV-filled terminal cytoplasm. With *in silico* modeling and protein lipid-binding assays, we demonstrate that an amphipathic liquid packing sensor motif (ALPS) at the N terminus of Piccolino binds SV-like liposomes, implicating this interaction as the mechanism underlying SV tethering. Together, our findings identified Piccolino as the molecular link between the SR and SVs.

## Teaser

How SRs tether hundreds of synaptic vesicles to sustain continuous neurotransmitter release at sensory synapses remains unknown. In the current study, Gierke et al. demonstrate that the SR-associated protein Piccolino is essential for this tethering and propose a direct protein–lipid interaction as the underlying molecular mechanism.

## Introduction

Neurons in the central nervous system communicate mainly via chemical neurotransmission at synapses. Chemical synapses exhibit remarkable diversity in the rate, speed, and duration of neurotransmitter release. Perhaps the most striking example of this diversity is the sensory synapse, which must be capable of continuous signal transmission over a wide range of stimulus intensities without fatigue. To meet this challenge, the sensory synapses of the photoreceptors and inner hair cells (IHCs) of the eye and ear, respectively, have evolved a specialized organelle, the synaptic ribbon (SR). It is an electron-dense, plate-like presynaptic structure that sits atop the active zone (AZ) and is thus considered an extension of the AZ. A hallmark of SRs is their surrounding halo of release-ready synaptic vesicles (SVs). At the base of SRs, SVs are closely associated with voltage-gated Ca^2+^ channels (tom Dieck et al., 2005) and fuse with the AZ upon Ca^2+^-triggered exocytosis. SVs located at the distal end of SRs rapidly replenish release sites, enabling coordinated and continuous neurotransmitter release (tom Dieck and Brandstätter, 2006; Regus-Leidig and Brandstätter, 2012; Moser et al., 2020). The molecules that govern SV binding to SRs and the subsequent transport to the AZ are, however, unknown. Insight into this process may be drawn from the better-characterized mechanisms of SV trafficking at conventional brain synapses. At conventional synapses, SVs are tethered directly to the AZ membrane through a tightly coordinated sequence of steps that prepare SVs for release. First, SVs are clustered near the AZ by tethering to the actin cytoskeleton via the phosphoprotein synapsin I (Bähler and Greengard, 1987; Benfenati et al., 1989). Next, SVs make contact with the AZ at distances of >100 nm (Cole et al., 2016). This restricts their free diffusion and brings them into proximity with the release site. This long-range AZ tethering step is thought to be mediated by the large AZ scaffold proteins Piccolo and Bassoon, though the precise molecular mechanism remains unknown. Shorter AZ tethers—mediated by a tripartite RIM–Rab3–Munc13 complex—then bring SVs within nanometers of Ca^2+^ channels for subsequent docking and priming (Fernández-Busnadiego et al., 2013; Dulubova et al., 2005). Thus, at conventional chemical synapses, AZ tethering designates the physical attachment of SVs to the AZ scaffold.

Ribbon synapses share the final AZ tethering step with conventional synapses, but differ in one critical respect: SVs do not proceed directly to the AZ. Instead, they are first captured by the SR, a step that has no counterpart at conventional synapses and is independent of synapsin I and actin (Mandell et al., 1990; Usukura and Yamada, 1987). SV tethering to the SR is achieved by fine filamentous tethers that extend perpendicularly from the SR surface and capture freely diffusible SVs from the terminals’ cytoplasm. Early EM studies suggested that individual SVs are connected to the ribbon by 3–5 tethers, each 30–50 nm in length (Usukura and Yamada, 1987). Electrophysiological studies further support the functional relevance of tethering, as the number of SVs released following a strong stimulus closely matches the number of SVs tethered to the SRs (Heidelberger et al., 1994; von Gersdorff et al., 1996). Moreover, the movement of tethered SVs along the SR toward the AZ is rate-limiting for SV release (Holt et al., 2004; Graydon et al., 2014). Together, these data indicate that tethering prepares SVs for release and dictates the rate of their descent along the ribbon toward the AZ—a mechanism that is thus central to the faithful encoding of sensory information.

Despite the importance of SV tethering for ribbon synapse function, the molecular identity underlying this process remains unknown. Here, we investigated the role of Piccolino, a splice variant of the AZ protein Piccolo and the largest known integral component of SRs (330 kDa; [Regus-Leidig et al., 2013]), as a candidate tethering molecule. We previously demonstrated that the C terminus of Piccolino interacts with RIBEYE, the major structural component of SRs (Schmitz et al., 2000; Müller et al., 2019). In the current study, we asked whether Piccolino could act as an SV tether in photoreceptor ribbon synapses. We demonstrate that Piccolino meets all requirements to qualify as an SV tethering molecule (Eisenberg-Bord et al., 2016).

## Results

### Generation and characterization of a conditional, rod photoreceptor-specific Piccolino knockout mouse line

**Figure 1.**
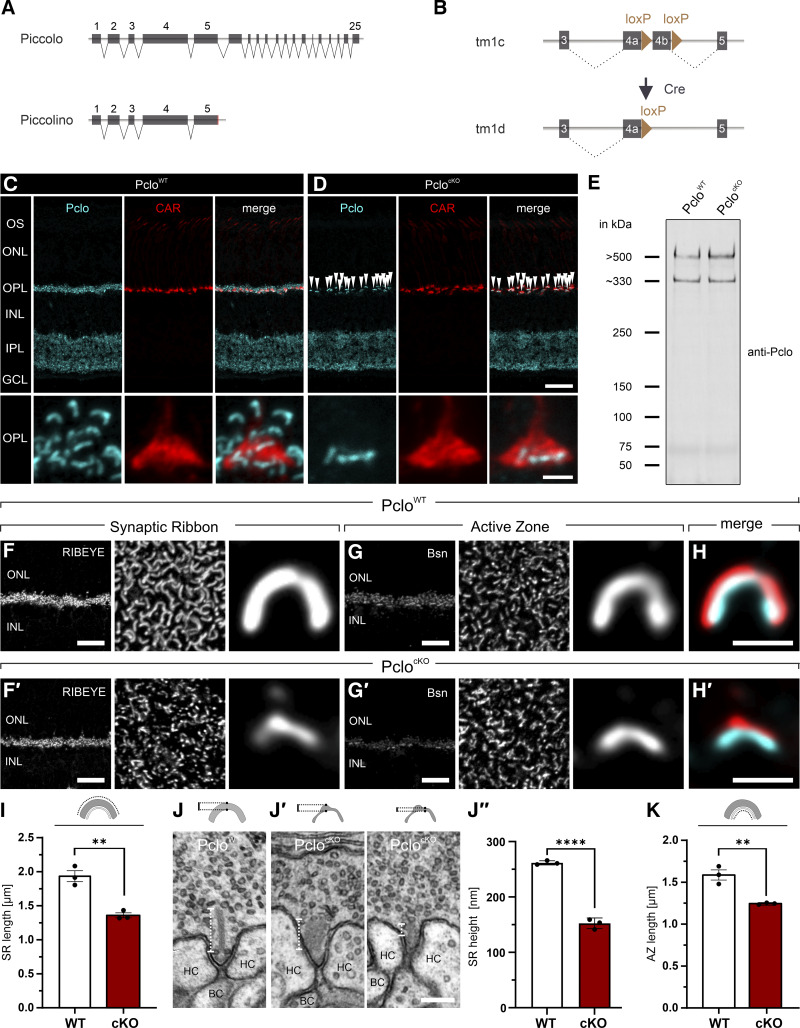
**Generation and characterization of a rod photoreceptor–specific Pclo knockout mouse line (Pclo** ^**cKO**^ **). (A)** Exon-intron structure of the *Pclo* gene indicating splicing of full-length Piccolo (top) and Piccolino (bottom). **(B)** Schematic representation of the genetic strategy used for generating Pclo^cKO^ ms. **(C and D)** Fluorescence micrographs of vertical cryostat sections through the retinae of Pclo^WT^ (C) and Pclo^cKO^ ms (D) double-labeled for Pclo (cyan) and cone arrestin (CAR, red). Arrowheads (➤) in D point to Pclo-positive cone photoreceptor terminals. **(E)** Western blot of whole retinal lysates of Pclo^WT^ and Pclo ^cKO^ synaptosomal fractions (P2) labeled with Pclo4 antibodies. **(F and F′)** CLSM images of vertical sections (left), whole-mount retina (middle), and a single SR (right) labeled against RIBEYE to measure SR length in Pclo^WT^ (F) and Pclo^cKO^ ms (F′). **(G and G′)** CLSM images of vertical sections (left), whole-mount retina (middle), and a single SR (right) labeled against Bassoon (Bsn) to measure AZ length in Pclo^WT^ (G) and Pclo^cKO^ ms (G′). **(H and H′)** Merge of Pclo^WT^ and Pclo^cKO^ SR and AZ stainings shown in F and G and F′ and G′, respectively. **(I)** Quantification of SR length in Pclo^WT^ and Pclo^cKO^ rod photoreceptors. Data are mean ± SD, **P = 0.0029, unpaired *t* test, *n* = 100 ribbons per animal, three animals. **(J and J′)** Representative EM micrographs of Pclo^WT^ (J) and Pclo^cKO^ (J′) rod photoreceptor SRs used for measuring SR height (dashed line). **(J″)** Quantification of rod photoreceptor SR height in Pclo^WT^ and Pclo^cKO^ retinae. Data are mean ± SEM, ****P < 0.0001, unpaired *t* test, 18–33 ribbons per animal, three animals. **(K)** Quantification of AZ length in Pclo^WT^ and Pclo^cKO^ rod photoreceptors. Data are mean ± SD, **P = 0.0051, unpaired *t* test, *n* = 100 ribbons per animal, three animals. Scale bar = 20 µm (overview) and 2 µm (high-power view) in D for C and D, 20 µm in F–G′; 1 µm in H and H′ for single-ribbon views in F, F', G, G′, H, and H′ and 0.5 µm in J′ for J and J″. loxP, locus of X-over P1; Cre, Cre-recombinase; OS, outer segments; ONL, outer nuclear layer; INL, inner nuclear layer; GCL, ganglion cell layer. Source data are available for this figure: SourceData F1.

To investigate the potential role of Piccolino as an SV tether in photoreceptor ribbon synapses, we generated a conditional, rod photoreceptor-specific Piccolino knockout mouse line (Pclo^cKO^). LoxP sites were inserted into the *Pclo* gene (Pclo^fl/fl^), followed by crossbreeding with a mouse line expressing Cre recombinase under the rhodopsin promotor (Rho-iCre; [Li et al., 2005]), which selectively knocks out the *Pclo* gene and thus Piccolino in rod photoreceptors (Fig. 1, A and B). To validate the selective knockout of Piccolino in rod photoreceptors, we labeled Pclo^WT^ and Pclo^cKO^ retinae with antibodies detecting all known Piccolo/Piccolino splice variants (Pclo 4 epitope [Spiwoks-Becker et al., 2008; Regus-Leidig et al., 2013]). In Pclo^WT^ retinae, strong Piccolino labeling was visible in both synaptic layers, the outer plexiform layer (OPL) and inner plexiform layer (IPL) (Fig. 1 C). In the OPL, Piccolino was present in rod and cone photoreceptor terminals, as best seen in the higher power views of the OPL in Fig. 1 C. In the OPL of Pclo^cKO^ retinae, Piccolino immunofluorescence was absent from rod photoreceptor terminals. Piccolino labeling was indistinguishable from WT controls in cone photoreceptor terminals labeled with an antibody against cone arrestin, as best seen in the higher power views of the OPL in Fig. 1 D. Previously, we found that a truncated Piccolino was still expressed in a Pclo^gt/gt^ rat (Müller et al., 2019). Such a fragment is theoretically possible from our cKO approach. To test for a putative rest fragment, we performed western blot analysis using Pclo^WT^ and Pclo^cKO^ whole retinal lysates using the Pclo4 antibody (Fig. 1 E). Both lysates showed a strong band at ∼550 kDa, corresponding to full-length Piccolo expressed at conventional chemical synapses in the IPL. Both lysates also showed a strong band at ∼330 kDa corresponding to Piccolino. Note that the remaining signal in Pclo^cKO^ lysates arises from the expression of Piccolino in cone photoreceptors and bipolar cells. No additional band was found in Pclo^cKO^ lysates, indicating that no truncated Piccolino is expressed.

**Figure S1.**
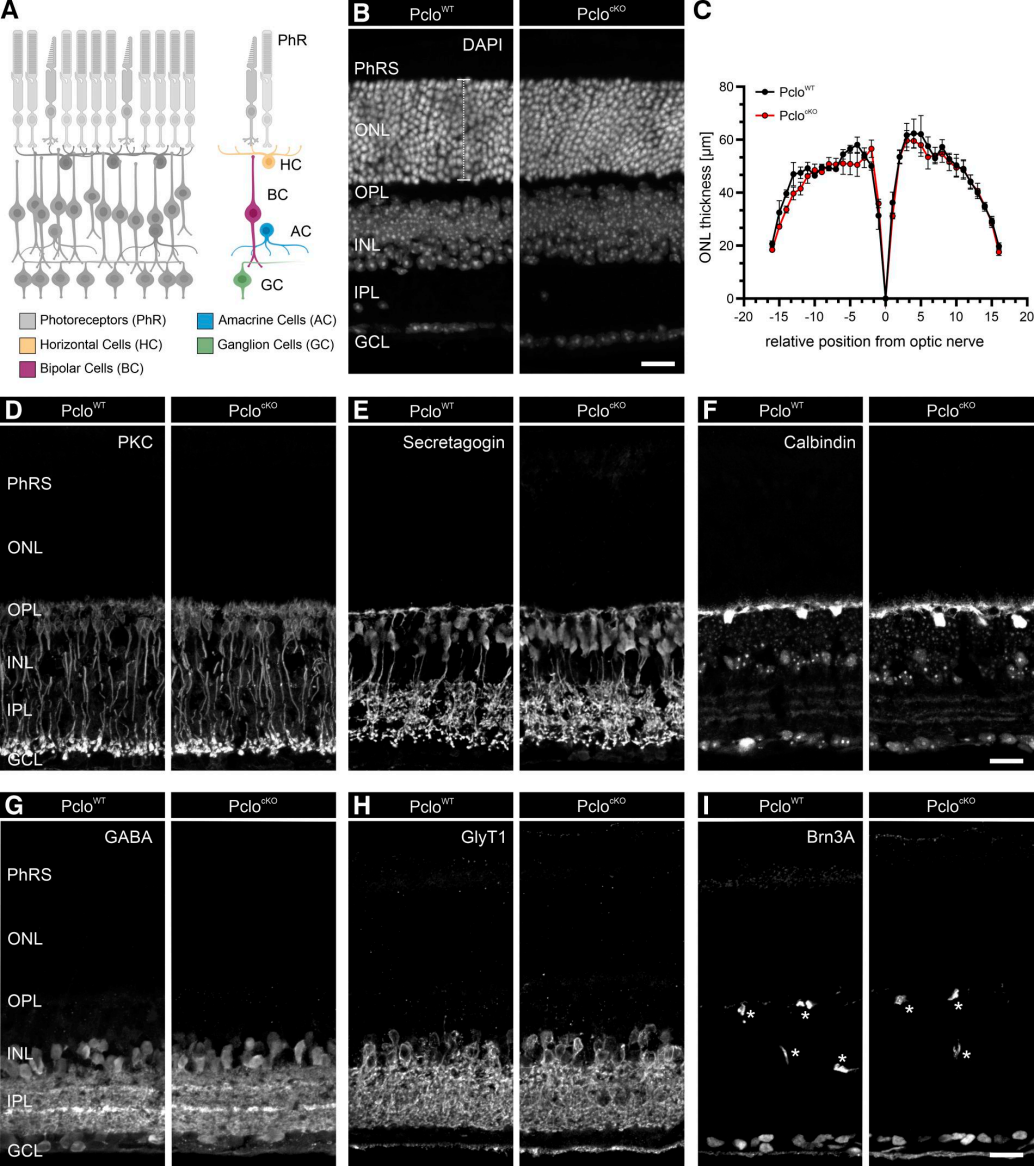
**Pclo** ^**cKO**^ **retinae exhibit normal retinal anatomy. (A)** Schematic representation of a mouse retina highlighting major neuronal cell classes. **(B)** Vertical cryostat sections through Pclo^WT^ and Pclo^cKO^ retinae labeled with DAPI. **(C)** Quantification of the ONL thickness in Pclo^WT^ and Pclo^cKO^ retinae. Data are mean ± SD, two-way ANOVA, four animals. **(D–I)** Fluorescence micrographs of vertical cryostat sections through Pclo^WT^ (left panels) and Pclo^cKO^ (right panels) retinae labeled for neuronal classes postsynaptic to rod and cone photoreceptors: rod bipolar cells (PKCα, D), cone bipolar cells (secretagogin, E), horizontal cells (calbindin, F), GABAergic amacrine cells (GABA, G), glycinergic amacrine cells (GlyT1, H), and ganglion cells (Brn3a, I). Asterisks (*) mark blood vessels. Scale bar = 20 µm in B, in F (for D–F), and in I (for G–I). PhRS, photoreceptor outer and inner segments; ONL, outer nuclear layer; OPL, outer plexiform layer; INL, inner nuclear layer; IPL, inner plexiform layer; GCL, ganglion cell layer.

Thus, the Pclo^cKO^ mouse line shows loss of Piccolino specifically in rod photoreceptor synapses. Piccolo/Piccolino expression in other neuronal classes of the retina, i.e., amacrine cells, cone photoreceptors, and bipolar cells, is not affected. Consistent with these findings, loss of Piccolino did not alter ONL thickness as a proxy for photoreceptor number (Fig. S1, A–C), or neuronal morphology (Fig. S1, D–I).

**Figure S2.**
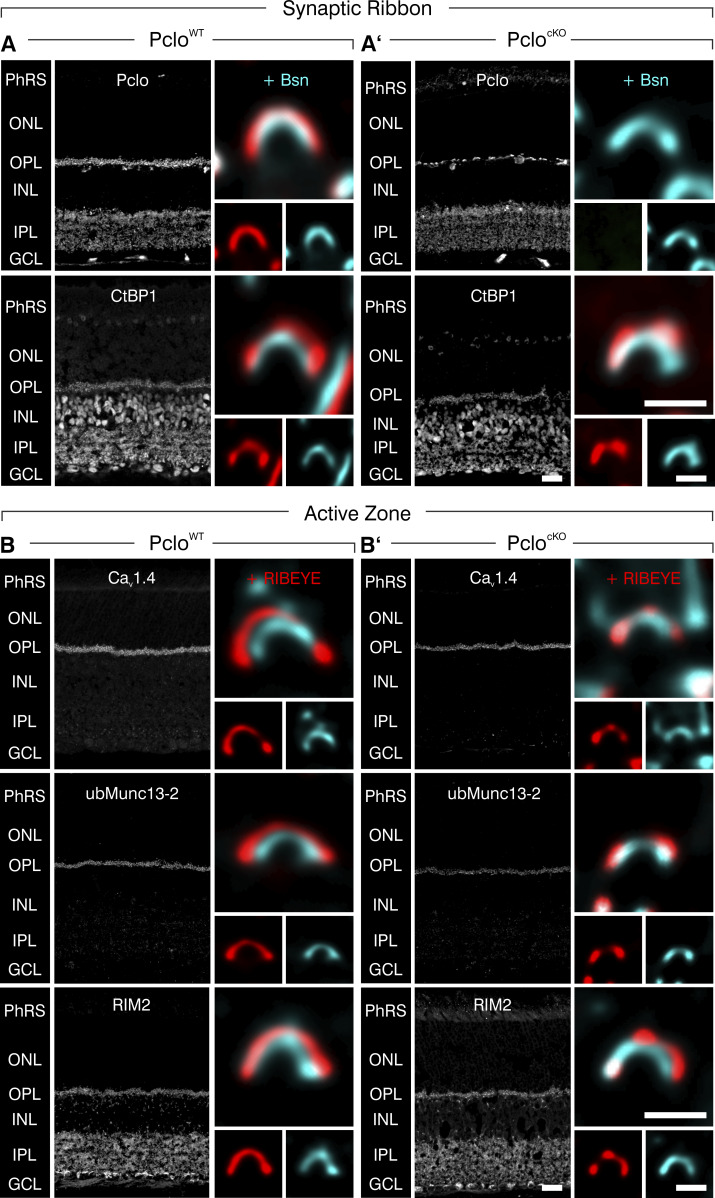
**Localization of SR and AZ proteins in Pclo** ^**WT**^ **and Pclo** ^**cKO**^ **retinae. (A and A′)** Left, CLSM images of vertical cryostat sections of Pclo^WT^ (A) and Pclo^cKO^ (A′) retinae labeled against the integral SR components Pclo and CtBP1. Right, High-power view of a single rod photoreceptor ribbon synapse labeled against the integral SR components, counterstained with Bsn to label AZs. **(B and B′)** Left, CLSM images of vertical cryostat sections of Pclo^WT^ (B) and Pclo^cKO^ (B′) retinae labeled against the integral AZ components Ca_v_1.4, ubMunc13-2, and RIM2α. *Right*, High-power view of a single rod photoreceptor ribbon synapse labeled against the integral AZ components, counterstained with RIBEYE to label SRs. Scale bar = 20 µm (overview) and 1 µm (high-power) in A′ and B′ (for A and B). Bsn, bassoon.

Importantly, the morphology and length of the SRs in the Pclo^cKO^ rod photoreceptors were reduced when compared with the Pclo^WT^ controls. As seen by RIBEYE labeling, SRs were no longer plate-shaped (Fig. 1, F, F′, and G′), and SR length and height were significantly reduced (Fig. 1, I and J–J″). The loss of the morphological integrity of Pclo^cKO^ rod photoreceptor SRs is in line with results of previous studies analyzing the effect of a shRNA knockdown of Piccolino in the mouse retina (Regus-Leidig et al., 2014) and the effect of loss of Piccolino in a Piccolo gene trap rat model (Müller et al., 2019). In this context, it is also interesting to note that the length of the AZ was reduced in Pclo^cKO^ rod photoreceptors (Fig. 1, G, G′, and K) and scaled with the length of the SR, as seen in double-labeling for Bassoon (AZ marker, [tom Dieck et al., 1998]) and RIBEYE (Fig. 1, H, H′, and I). Deletion of Piccolino did not affect the localization of other SR proteins (Fig. S2, A and A′) and AZ proteins (Fig. S2, B and B′).

### Loss of Piccolino ablates SV tethering in rod photoreceptor ribbon synapses

**Figure 2.**
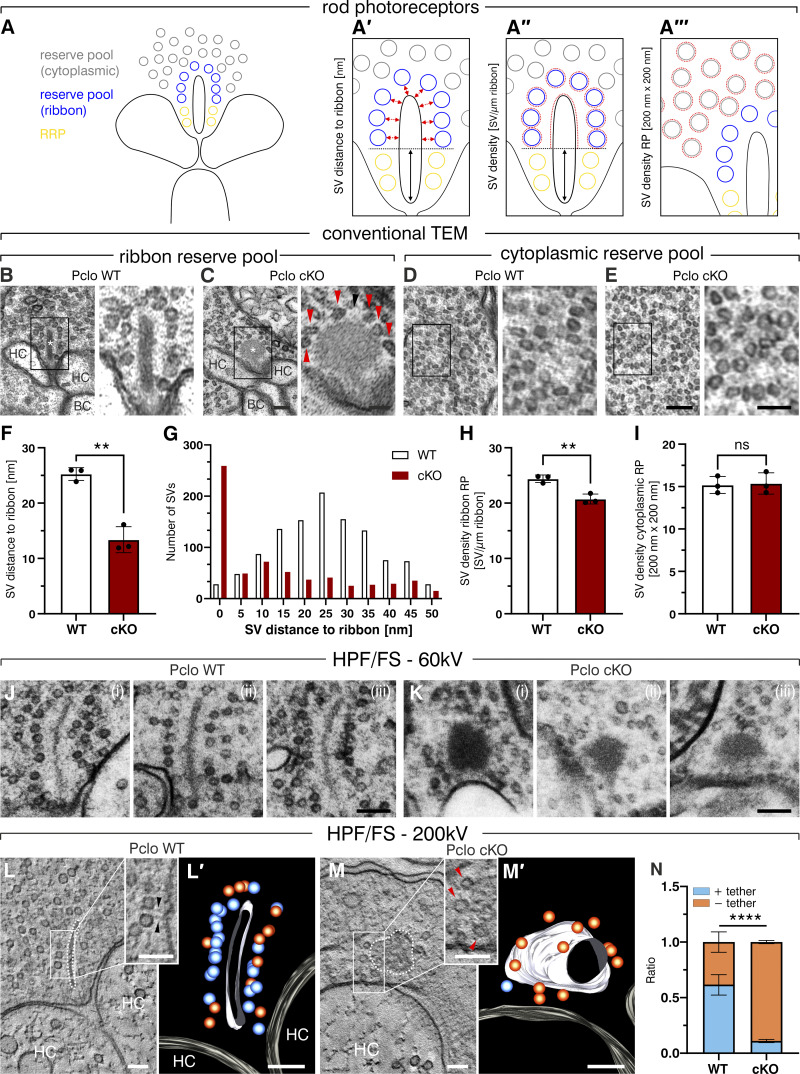
**Loss of Piccolino results in altered distance and density of SVs and ablates SV tethering at rod photoreceptor SRs. (A)** Schematic of a rod photoreceptor ribbon synapse with SV pools: RRP (yellow), ribbon RP (blue), and cytoplasmic RP (gray). **(A′–A‴)** Schematic representation of methodology used for measuring SV distance (A′) and SV density in the ribbon RP (A″) and the cytoplasmic RP (A‴). Quantified parameters are indicated by dashed red lines. **(B and C)** Representative EM micrographs of Pclo^WT^ (B) and Pclo^cKO^ (C) rod photoreceptor SRs. Red arrowheads (➤) indicate SVs in direct contact with the SR surface; a black arrowhead (➤) indicates a disrupted SV halo. **(D and E)** Representative EM micrographs of SVs in Pclo^WT^ (D) and Pclo^cKO^ (E) rod photoreceptor terminals. **(F and G)** Quantification (F) and frequency distribution (G) of the distance between SVs in the ribbon RP and the SR in Pclo^WT^ and Pclo^cKO^ rod photoreceptors. Data are mean ± SD, **P = 0.0014, unpaired *t* test, *n* = 66–78 terminals per animal, three animals. **(H)** Quantification of SV density in the ribbon RP (SVs per µm SR) in Pclo^WT^ and Pclo^cKO^ rod photoreceptors. Data are mean ± SD, **P = 0.0050, unpaired *t* test, *n* = 66–78 terminals per animal, three animals. **(I)** Quantification of SV density in the cytoplasmic RP (SVs per 0.04 µm^2^) in Pclo^WT^ and Pclo^cKO^ rod photoreceptors. Data are mean ± SD, n.s., P = 0.8579, unpaired *t* test, *n* = 62–74 terminals per animal, three animals. **(J and K)** Representative conventional TEM micrographs of HPF/FS-prepared Pclo^WT^ (J, i–iii) and Pclo^cKO^ (K, i–iii) rod photoreceptor terminals, confirming recapitulation of the immersion-fixed phenotype: a halo of SVs at a distance from the SR in Pclo^WT^ and its absence in Pclo^cKO^. **(L–M′)** Representative EM tomogram virtual sections and 3D reconstructions of a Pclo^WT^ (L and L′) and a Pclo^cKO^ rod photoreceptor SR (M and M′). **(N)** Quantification of SVs connected (+tether, blue) or not connected (−tether, orange) to SRs in Pclo^WT^ and Pclo^cKO^ rod photoreceptors. Data are ± SD, ****P < 0.0001, unpaired *t* test, *n* = 10 tomograms for each genotype, two animals. Scale bar = 200 nm in C for B and C and in E for D and E, 100 nm in high power views and in L–M′. HC, horizontal cell; BC, bipolar cell.

Next, we examined whether loss of Piccolino affects the organization of the ribbon-associated SVs, which are assigned to two distinct SV pools (Thoreson, 2021) (Fig. 2 A): a readily releasable SV pool (RRP), representing the bottom two rows of SVs docked to the base of photoreceptor SRs, and a reserve pool of SVs (ribbon RP), representing the SVs tethered higher up on the SR. The ribbon-associated SVs are replenished from a third SV pool, the cytoplasmic, freely diffusible pool of SVs (cytoplasmic RP), which comprises several thousand SVs in mouse rod photoreceptor terminals (Zampighi et al., 2011).

We first analyzed, whether the distance of individual SVs to SRs, which is dictated by tethering molecules, was different between Pclo^WT^ and Pclo^cKO^ rod photoreceptors (Fig. 2 A′). Quantitative EM revealed that in Pclo^WT^ rod photoreceptor ribbon synapses, SVs of the ribbon RP were located at a mean distance of 25.25 ± 0.96 nm from the SR (Fig. 2, B and F). This distance was significantly reduced in Pclo^cKO^ rod photoreceptor ribbon synapses with a mean distance of 14.39 ± 1.66 nm (Fig. 2, C and F). Frequency distribution analysis further revealed that in Pclo^WT^, the majority of SVs of the ribbon RP were located between 15 and 35 nm from the SR (Fig. 2 G, white bars), whereas in Pclo^cKO^, a large fraction of SVs was located <5 nm from the SR (Fig. 2 G, red bars).

Next, we analyzed the SV density in the ribbon RP in single ultrathin sections. We traced the length of individual SRs and counted all SVs within a distance of <50 nm of the SRs (Fig. 2 A″). In Pclo^WT^ rod photoreceptor ribbon synapses, the density of SVs in the ribbon RP was 24.4 ± 0.56 SVs/µm SR (Fig. 2, B and H). In Pclo^cKO^ rod photoreceptor ribbon synapses, the SV density in the ribbon RP was significantly reduced to 20.73 ± 0.73 SVs/µm (Fig. 2, C and H). To exclude that the reduced density of SVs in the ribbon RP in Pclo^cKO^ rod photoreceptors was caused by a reduced number of SVs in the cytoplasmic RP, which replenishes the ribbon RP, we compared the SV density in the cytoplasmic RP between Pclo^WT^ and Pclo^cKO^ (Fig. 2 A‴). SV density in the cytoplasmic RP was not significantly different between Pclo^WT^ and Pclo^cKO^ rod photoreceptor terminals (Pclo^WT^: 15.19 ± 1.00 SVs/0.04 µm^2^; Pclo^cKO^: 15.37 ± 1.26 SVs/0.04 µm^2^; Fig. 2, D, E, and I).

To directly visualize SR-associated tethers, tissue was prepared by high-pressure freezing and freeze substitution (HPF/FS) to optimally preserve ultrastructure (Chakrabarti et al., 2018; Michanski et al., 2023). For quality control, we first analyzed HPF-treated tissue with conventional TEM using ultrathin sections. HPF-treated tissue closely recapitulated the findings from immersion-fixed tissue: in Pclo^WT^ rod photoreceptors, a halo of SVs at a distance from the SR was observed (Fig. 2, J (i–iii)), whereas this halo was absent in Pclo^cKO^ rod photoreceptors (Fig. 2, K (i–iii)). Having confirmed that HPF/FS faithfully preserved ultrastructure, we next switched to 200 kV transmission electron tomography to resolve individual tethers. We reconstructed Pclo^WT^ and Pclo^cKO^ rod photoreceptor SRs in 3D and analyzed tethers of SVs in the ribbon RP. The SVs were subdivided into tethered (+ tether) and non-tethered (– tether). In Pclo^WT^ rod photoreceptors, 60.6 ± 9.52% of all SVs in the ribbon RP within 50 nm of the SR were connected to the SR via tethers (Fig. 2 L, black arrowheads, Fig. 2, L′ and N), whereas 39.20 ± 9.52% of SVs were not (Fig. 2, L, L′, and N). In Pclo^cKO^ rod photoreceptors, in contrast, only 11.3 ± 5.73% of all SVs in the ribbon RP within 50 nm of the SR were connected via tethers, whereas 88.7 ± 5.73% of SVs were not (Fig. 2, M, M′, and N). Notably, we frequently observed SVs in direct physical contact with the SR surface in Pclo^cKO^ rod photoreceptors (Fig. 2 M, red arrowheads), in line with observations from conventional TEM (Fig 2, C and G).

Taken together, our data show that the loss of Piccolino from rod photoreceptors results in a significant reduction in the SV distance and density in the ribbon RP due to the almost complete loss of SV tethers.

### Nanoscale localization of Piccolino at rod photoreceptor ribbon synapses

**Figure 3.**
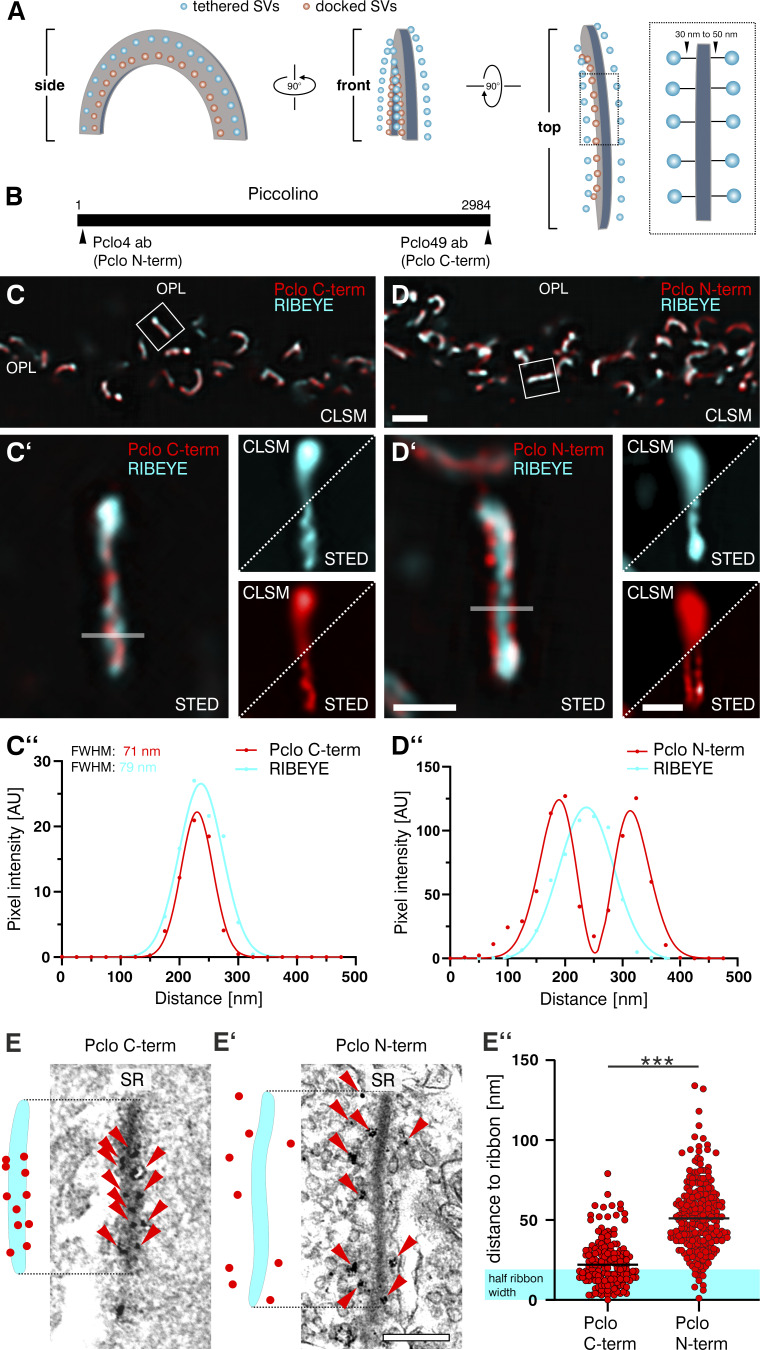
**Piccolino is oriented at rod photoreceptor synapses. (A)** Schematic 3D representation of a rod photoreceptor SR viewed from the side, front and top. **(B)** Schematic representation of Piccolino indicating binding sites for Pclo4 (Pclo N-term) and Pclo49 (Pclo C-term) antibodies. **(C and D)** Representative fluorescence micrographs of WT retinae double-labeled with Pclo C-term (C) and Pclo N-term (D) antibodies co-stained with the SR marker RIBEYE. **(C′ and D′)** High-power views of STED microscopy images of a single SR viewed from the top labeled with Pclo C-term (C′) and Pclo N-term (D′) antibodies co-stained with the SR marker RIBEYE. **(C″ and D″)** Line intensity profiles corresponding to lines in C′ and D′. **(E and E′)** Representative pre-embedding immunoelectron micrographs of rod photoreceptor SRs labeled with Pclo C-term (E) and Pclo N-term (E′) antibodies. Arrowheads (➤) point to individual immunogold puncta. **(E″)** Quantification of distance for individual immunogold puncta to the center of SRs. Data are *n* = 157 puncta from three individual experiments (Pclo C-term) and *n* = 262 puncta from four individual experiments (Pclo N-term). Mean values were compared using an unpaired *t* test, ***P < 0.001. Scale bar = 2 µm in D for C and D, 0.5 µm in D′ for C′ and D′ and 0.2 μm in E′ for E and E′. AU, arbitrary units; ab, antibody; FWHM, full-width at half maximum.

Rod photoreceptor SRs are 3D objects that appear horseshoe-shaped when viewed from the side and ribbon-shaped when viewed from the front or the top (Fig. 3 A). As seen in the front and top view, tethers extend perpendicular from the SR into the terminals’ cytoplasm, where they hold SVs at a distance of ∼ 30–∼ 50 nm (Usukura and Yamada, 1987) (Fig. 3 A). Consequently, we investigated whether Piccolino is able to span a distance of ∼ 30–∼ 50 nm from the SR. For this, we analyzed the *in situ* topology of Piccolino at the SR by STED and immuno-EM using two antibodies that recognize epitopes at the two opposite ends of Piccolino: N terminus: Pclo4 antibody, AA E^74^-A^260^ (Spiwoks-Becker et al., 2008); C terminus: Pclo49, AA G^2962^-G^2984^ (Regus-Leidig et al., 2013) (Fig. 3 B). While CLSM images show a colocalization of both Pclo antibodies with RIBEYE (SR marker) with no apparent differences between the staining patterns (Fig. 3, C and D), STED microscopy resolved a striking difference in the *in situ* topology of the two Pclo antibodies at the SR (Fig. 3, C′ and D′). The Pclo C-terminal antibody labeling overlapped with the RIBEYE labeling (Fig. 3, C′, and C″), whereas the Pclo N-terminal antibody labeling flanked the RIBEYE labeling on each side (Fig. 3 D′ and D″). This provides the first evidence that the N terminus of Piccolino is located at some distance from the SR.

To analyze the distance between the C- and N-terminal epitopes of Piccolino and the SR with highest spatial resolution, we next performed immuno-EM. We measured the distance of Pclo C- and Pclo N-terminal–positive immunogold puncta from the SR, which is visible as an electron-dense structure in EM, in single ultrathin sections of WT rod photoreceptor ribbon synapses. Pclo C-terminal–positive puncta were almost exclusively located directly on the SR and were rarely detected in the cytoplasm (Fig. 3 E, red arrowheads). The average distance between Pclo C-terminal–positive puncta and the SR center was 24 ± 14 nm (Fig. 3 E″). This positions the C terminus of Piccolino within 10 nm of the SR, considering a SR width of ∼ 30 nm (or ∼ 15 nm from the SR center). In contrast, Pclo N-terminal–positive puncta were located away from the SR center in the cytoplasm (Fig. 3 E′, red arrowheads). The average distance between Pclo N-terminal–positive puncta and the SR center was 52 ± 22 nm, which was significantly different from the location of Pclo C-terminal–positive puncta (Fig. 3 E″).

In summary, the results from STED and immuno-EM revealed the nanoscale topology of Piccolino at rod photoreceptor SRs: the C terminus is directed toward the SR, while the N terminus extends ∼ 30 nm away from the SR into the cytoplasm, where it could interact with the SVs.

### An ALPS motif at the N terminus of Piccolino is necessary and sufficient to bind SV-like liposomes *in silico* and *in vitro*

Next, we investigated the molecular basis of Piccolino’s interaction with SVs. In our search for a potential interacting motif, we found a publication predicting an amphipathic lipid packing sensor (ALPS) motif at the N terminus of Piccolino (AAs V^865^-L^883^, see Table 1 in Drin et al. [2007]). ALPS motifs mediate, among others, tethering at the Golgi apparatus. Interestingly, ALPS motifs do not bind to a specific lipid, but exploit packing defects, i.e., regions in lipid bilayers with transiently exposed lipid tails, which arise from lipid headgroup geometry, unsaturation, or membrane curvature (Antonny, 2011; Giménez-Andrés et al., 2018).

**Figure 4.**
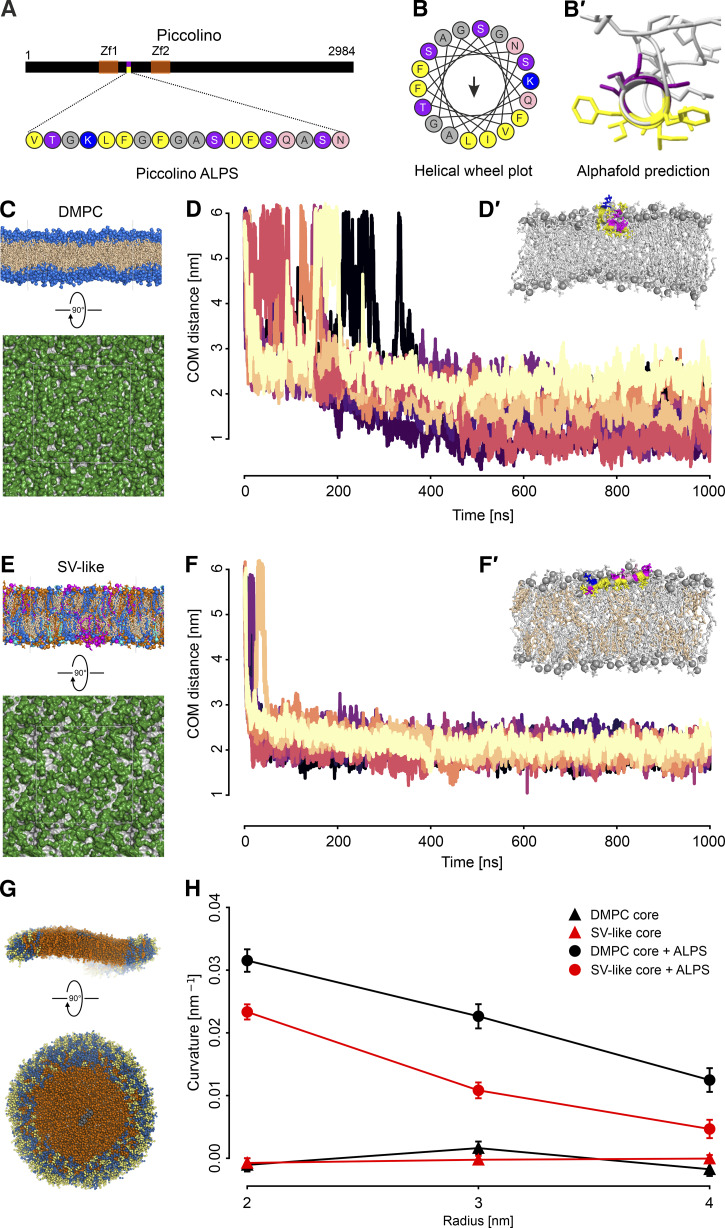
**Piccolino ALPS binds to membranes in atomistic and CG MD simulations. (A)** Schematic of Piccolino (black) including the location of the ALPS motif (yellow/purple). **(B and B′)** 2D helical wheel plot representation of the α-helical Pclo ALPS motif with color-coded amino acids (B) and 3D structure of the Pclo ALPS motif and its surrounding amino acids (B′) predicted using AlphaFold. AAs are color-coded according to their biochemical properties: strongly hydrophobic (yellow), weakly hydrophobic (gray), polar uncharged (purple, red), and basic (blue). Arrow (→) in B indicates hydrophobic moment. **(C)** Side and top view of a simulated (all-atom) DMPC membrane. The top view shows the hydrophobic defects (gray) and the hydrophilic heads (green) with hydrophilic heads (blue) and hydrophobic tails (wheat). **(D and D′)** Center of mass (COM) distance between the Piccolino ALPS motif and a DMPC membrane (D) and snapshot of the simulation with the ALPS peptide (D′). PLs are colored in light gray and the ALPS peptide as in A. **(E)** Side and top view of a simulated (all-atom) SV-like membrane. The lipids are colored as follows: CHOL (wheat), DOPE (blue), SM18 (cyan), DOPS (magenta), and POPC (orange). The top view shows the hydrophobic defects (gray) and the hydrophilic heads (green). **(F and F′)** COM distance between the Piccolino ALPS motif and an SV-like membrane (F) and snapshot from a single SV-like membrane simulation with the ALPS motif (F′). Color code as in B and CHOL in wheat. **(G)** Bicelle simulation with an SV-like core and a Piccolino ALPS motif in side and top view. Color code for structures: ALPS peptide (dark gray), SV-like core lipids (orange), long-chained rim lipids (DOPC, blue), and short-chained lipids (DTPC, yellow). **(H)** Mean locally induced curvature by the Piccolino ALPS motif as a function of the peptide COM distance for DMPC core (black) and SV-like core (red). Triangles (▲) represent plain bicelles, circles (●) represent bicelles with the ALPS motif.

Notably, the Pclo ALPS motif displays amphipathic characteristics. It is enriched in polar serine and threonine residues and bulky hydrophobic side chains, while it is depleted in charged residues (Fig. 4, A, B, and B′). To test whether this AA composition renders the motif sensitive to a specific lipid composition, we performed µs-long, all-atom and coarse-grained (CG) molecular dynamics (MD) simulations. We focused on the spontaneous membrane binding of the ALPS motif to two distinct lipid bilayer systems: a symmetric 1,2-dimyristoyl-sn-glycero-3-phosphocholine (DMPC) bilayer with a small fraction of exposed hydrophobic surface area (0.15 ± 0.06%, Fig. 4 C) and a SV-mimetic membrane composed of cholesterol (CHOL) 1,2-dioleoyl-sn-glycero-3-phosphoethanolamine (DOPE), 1-palmitoyl-2-oleoyl-sn-glycero-3-phosphocholine (POPC), 1,2-dioleoyl-sn-glycero-3-phospho-L-serine (DOPS), and N-stearoyl-D-erythro-sphingosylphosphorylcholine (SM18) (40:25:22:8:5, mol %) with a markedly higher fraction of exposed hydrophobic surface area (2.92 ± 0.57%, Fig. 4 E). The ALPS motif of Piccolino (AAs V^865^-L^883^) was initially modeled in an α-helical conformation. In all simulations, the Pclo ALPS motif bound spontaneously to the DMPC lipid bilayer interface with occasional unbinding (<400 ns) (Fig. 4, D and D′). In contrast, binding to the SV-like membrane occurred significantly faster (<50 ns) compared with the symmetric DMPC bilayer and no unbinding was observed over the course of the simulation (Fig. 4, F and F′). This accelerated association is consistent with previous findings showing facilitated membrane insertion of ALPS motifs into membranes exhibiting more pronounced lipid bilayer defects (Vanni et al., 2013).

Lipid bilayer defects are governed not only by lipid composition but also by the degree of membrane curvature. Due to the high sensitivity of the ALPS motif to such defects, it is ideally suited as a curvature sensor and may contribute to vesicle recognition and tethering. To investigate this behavior further, we performed CG MD simulations to study the formation of ALPS-induced curvature via the spontaneous interaction of the ALPS motif with symmetric lipid bicelle systems (Fig. 4 G). In contrast to infinite periodic membranes, bicelles enable the unbiased study of protein-induced membrane deformation. The curvature induced by the Pclo ALPS motif was quantified as a function of distance from the binding site for both DMPC and SV-like bicelles (Fig. 4 H). The motif induced a pronounced local curvature, with a mean value of 0.032 ± 0.002 nm^−1^ for the DMPC bicelle (corresponding to a radius of 31.7 ± 1.8 nm) and 0.023 ± 0.001 nm^−1^ for the SV-like bicelle (corresponding to a radius of 42.9 ± 2.2 nm) within a 2 nm environment of the Pclo ALPS motif. This ALPS-binding–induced curvature marks an upper estimate for the optimal radius for ALPS-binding to the precurved membrane of SVs. Accordingly, the latter value is double the average SV diameter of 41.6 ± 8.4 nm as reported by (Takamori et al., 2006).

**Figure 5.**
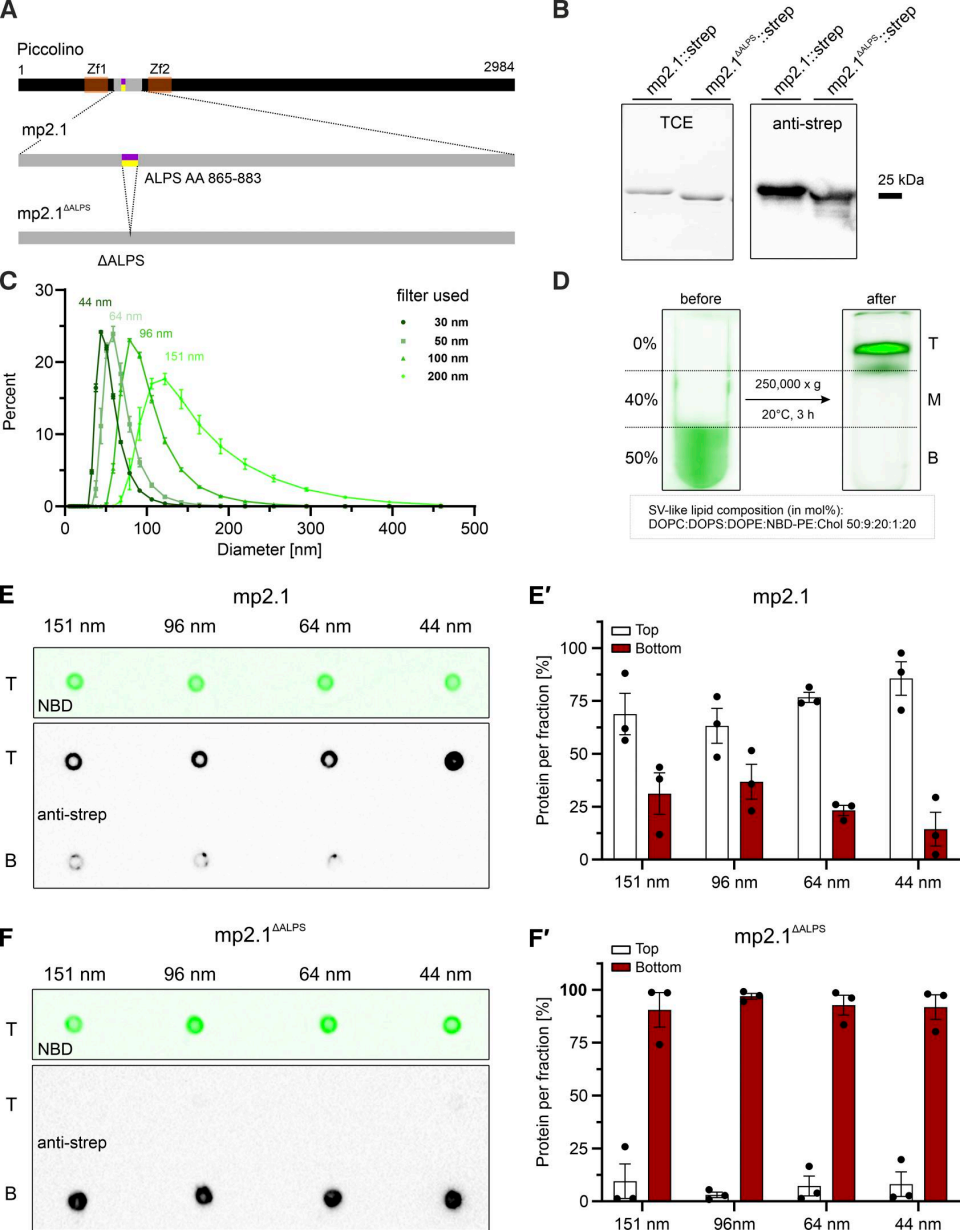
**An N-terminal Piccolino ALPS motif binds to artificial, SV-like liposomes. (A)** Schematic of Piccolino (black) including the location of the ALPS motif (yellow/purple) within the region used for protein–lipid binding studies (mp 2.1; gray). WT mp 2.1 without the ALPS motif (mp2.1^ΔALPS^) was used as a negative control. **(B)** SDS-PAGE with purified Pclo fragments labeled with the total protein stain TCE (left). Western blots of purified Pclo fragments mp2.1::strep and mp2.1^ΔALPS^::strep fragments stained with anti-strep (right). **(C)** Diameter of SV-like liposomes measured by DLS after extrusion through polycarbonate filters. **(D)** Detection of NBD-labeled lipids in centrifuge tubes with sucrose step gradients (0–50%) before (left) and after (right) ultracentrifugation. **(E–F′)** Dot blots of collected fractions (T, top; B, bottom) after liposome floatation assays and quantification of bound protein for WT mp2.1::strep (E and E′) and mutant mp2.1^ΔALPS^::strep (F and F′). The graphs in C, E′, and F′ show the mean values of *n* = 3 individual experiments. mp, mouse Piccolino; NBD, nitrobenzoxadiazole; Zf, zinc finger. Source data are available for this figure: SourceData F5.

**Figure S3.**
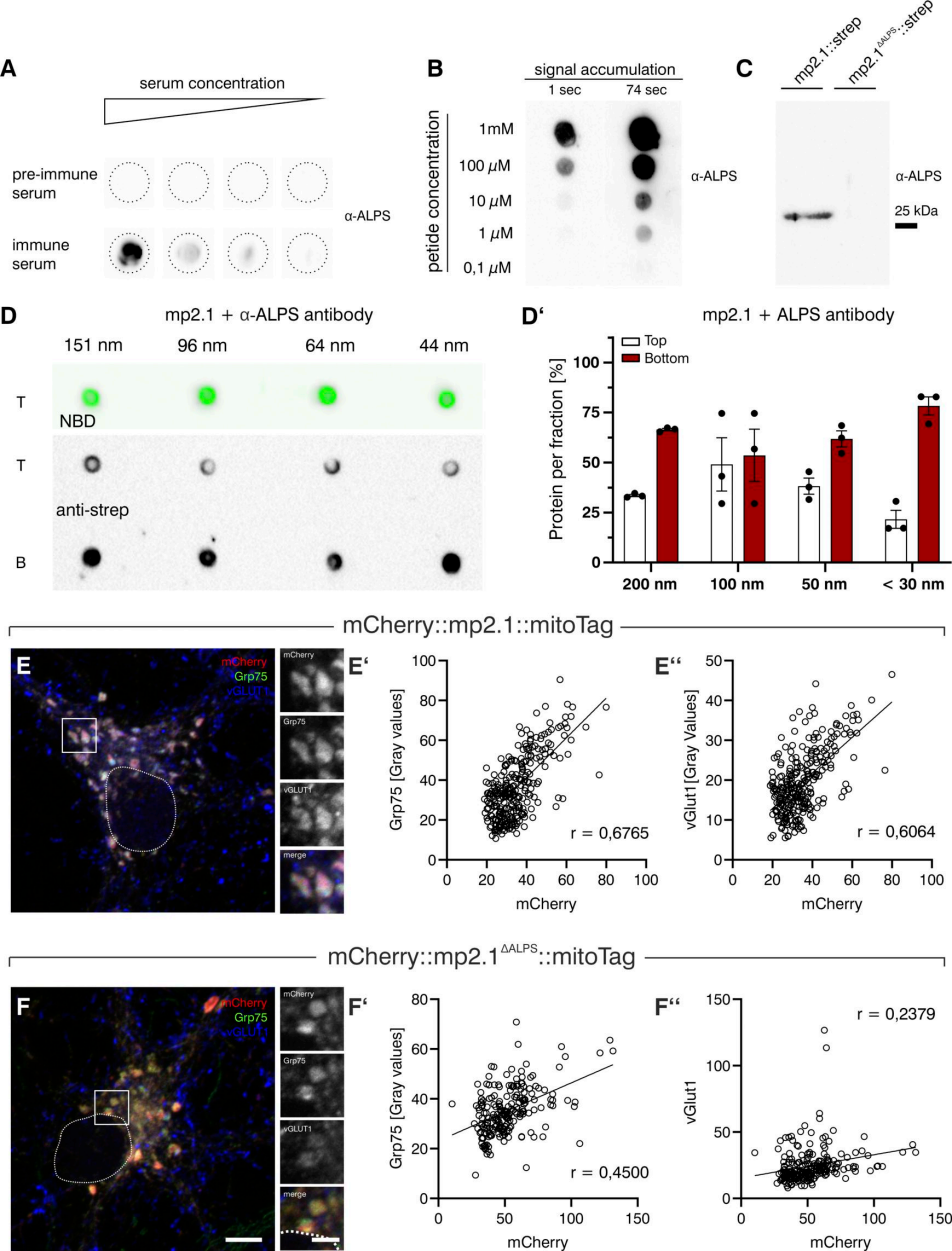
**Binding of the Piccolino ALPS motif to SV-like liposomes and endogenous SVs. (A)** Dot blot showing the presence of anti-Piccolino ALPS antibodies in immune sera before and after immunization. **(B)** Sensitivity of the anti-Piccolino ALPS antibody to different concentrations of ALPS peptide. Same dot blot imaged with different exposure times. **(C)** Western blot of purified Pclo fragments mp2.1::strep and mp2.1^ΔALPS^::strep protein stained with anti-Piccolino ALPS antibodies. **(D and D′)** Dot blots of collected fractions (T, top; B, bottom) after liposome floatation assays and quantification of bound protein for WT mp2.1::strep preincubated with anti-Piccolino ALPS antibodies. *n* = 3 individual experiments. mp, mouse Piccolino; NBD, nitrobenzoxadiazole. **(E and F)** Representative CLSM images of cultured cortical neurons infected with mCherry::mp2.1::mitoTag (E) or mCherry::mp2.1^ΔALPS^::mitoTag (red) (F) and immunolabeled for endogenous Grp75 (green) and vGLUT1 (blue). Dashed lines delineate the nucleus. **(E′ and F′)** Pearson’s *r* between endogenous Grp75 and mCherry::mp2.1::mitoTag (E′) or mCherry::mp2.1^ΔALPS^::mitoTag (F′) immunofluorescence signals as a measure of targeting efficiency. **(E″ and F″)** Pearson’s *r* between endogenous vGLUT1 and mCherry::mp2.1::mitoTag- (E″) or mCherry::mp2.1^ΔALPS^::mitoTag (F″) immunofluorescence signals as a measure of SV recruitment. *n* = 268 mitochondria from (eight cells, mCherry::mp2.1::mitoTag) and *n* = 216 mitochondria from (eight cells, mCherry::mp2.1^ΔALPS^::mitoTag). Scale bar = 5 µm (overview) and 2 µm (high-power view) in F for E and F. Source data are available for this figure: SourceData FS3.

To experimentally support the results of the MD simulations, we performed liposome floatation assays to test the potential direct interaction between the Pclo ALPS motif and artificial lipid vesicles. We mixed liposomes with an SV-like lipid composition consisting of the four major SV lipid components 1,2-dioleoyl-sn-glycero-3-phosphocholine (DOPC), DOPS, DOPE, and CHOL with recombinant proteins covering the N-terminal sequence between the two zinc finger domains of Piccolino flanking the ALPS motif (mp2.1, AAs T^733^-P^987^, Fig. 5 A). An identical fragment with a deletion of the ALPS motif (mp2.1^ΔALPS^) served as a negative control. The strep-tag II was fused to both proteins to enable affinity purification following expression in *Escherichia coli*. Successful overexpression and purification were validated by total protein stain (Fig. 5 B, 2,2,2-trichlorethanol [TCE] and by western blotting (WB) using an antibody directed against the strep-tag II (Fig. 5 B, anti-strep). Because MD simulations indicated potential curvature-dependent binding of the ALPS motif, mp2.1::strep recombinant proteins were mixed with NBD-labeled, SV-like liposomes of different diameters via extrusion through polycarbonate filters of different sizes (30–200 nm). Actual diameters of SV-like liposomes were verified using dynamic light scattering (DLS) (Fig. 5 C). As expected, the diameters of SV-like liposomes decreased with decreasing pore size (Fig. 5 C). Next, purified protein and SV-like liposomes were mixed, adjusted to 50% sucrose and overlaid with a 0%/40% sucrose step gradient prior to centrifugation (Fig. 5 D). After centrifugation, the lipid migration was confirmed by NBD fluorescence (Fig. 5, E and F, upper panels). Dot blot analysis revealed that the majority of the ALPS-containing mp2.1::strep protein was recovered at the 0%/40% interface, indicating its association with floating liposomes (Fig. 5 E, lower panel). The association appeared to be most efficient when small liposomes (30 nm filter) were used for the floatation assay (Fig. 5, E and E′). In contrast, the mutant mp2.1^ΔALPS^::strep protein showed a significantly reduced recovery in the top fraction (P < 0.0001 for all liposome sizes) and was predominantly detected at the bottom of the gradient (Fig. 5, F and F′). This indicates that the ALPS motif in mp2.1 is the site of protein–lipid binding. Notably, masking the mp2.1::strep protein with a custom antibody raised against the ALPS motif (Fig. S3, A–C) prior to centrifugation attenuated the binding of mp2.1::strep to SV-like liposomes to <50% (Fig. S3, D and D′).

We next asked whether mp2.1 can recruit native SVs in a cellular context. To this end, cultured cortical neurons were infected with AAV particles encoding mCherry::mp2.1::mitoTag or the deletion mutant mCherry::mp2.1^ΔALPS^::mitoTag. Infected neurons were triple-labeled for mCherry (endogenous signal), Grp75 (mitochondrial marker), and vGLUT1 (SV marker) and imaged by confocal microscopy (Fig. S3, E and F). Co-localization of Grp75 with mCherry served as a readout for targeting efficiency, and co-localization of Grp75 with vGLUT1 as a readout for SV recruitment. Both constructs showed efficient mitochondrial targeting, though slightly less so for the mutant (mp2.1: r = 0.6765; mp2.1^ΔALPS^: r = 0.4500; Fig. S3, E′ and F′). Strikingly, co-localization of mCherry with vGLUT1 was markedly reduced for mCherry::mp2.1^ΔALPS^::mitoTag compared with WT (mp2.1: r = 0.6064; mp2.1^ΔALPS^: r = 0.2379; Fig. S3, E″ and F″). This shows that mp2.1 is capable of recruiting SVs ectopically to mitochondria and that the ALPS motif is required for this recruitment, supporting a direct role of the Pclo ALPS motif in SV binding.

In summary, the results of the *in silico* MD simulations and the liposome flotation and SV recruitment assays suggest that the Pclo ALPS motif can interact with lipid bilayers via packing defects and that the N-terminal ALPS motif-containing Piccolino fragment mp2.1::strep is capable of associating with liposomes of SV-like composition and size in vitro and with native SVs *in vivo*.

## Discussion

Vesicle tethering is required in intracellular organelles to regulate cargo selection, vesicle positioning and fusion (Whyte and Munro, 2002; Brown and Pfeffer, 2010; Yu and Hughson, 2010). At chemical synapses, and particularly at high-throughput ribbon synapses, tethering is important for the recruitment of SVs to the presynaptic AZ, the site of fusion. Although ribbon-tethered SVs have been observed in photoreceptor ribbon synapses since the dawn of EM (Dowling and Boycott, 1966), the molecular identity of the tethers is still unknown. Here, we present several lines of evidence that Piccolino, the C-terminally truncated splice variant of the AZ protein Piccolo expressed at conventional synapses, tethers SVs to the SRs in photoreceptor ribbon synapses.

### Loss of Piccolino perturbs SV tethering in rod photoreceptor ribbon synapses

Strong evidence for Piccolino as an SV tether comes from the findings from the Pclo^cKO^ mouse line. Loss of Piccolino function significantly decreases SV density and distance at SRs in rod photoreceptors (see Fig. 2). These findings are reminiscent of “category2” IHC ribbons that were described in a Piccolino gene-trap knockout rat line (Michanski et al., 2023). Category2 IHC ribbons showed a loss of SVs specifically at the distal end of SRs. Despite this phenotype, the authors ruled out a role of Piccolino in SV tethering since ∼30% of all Pclo-deficient IHC ribbons examined were unaffected by the loss of Piccolino (“category1”) (Michanski et al., 2023). A possible explanation for this discrepancy between the SV tethering defect observed here and in the Michanski et al. (2023) study could be a variable efficiency of Piccolino deletion in individual cells between the knockout mouse model used here and the previously studied gene-trap knockout rat model. Consistent with this interpretation, a previous study from our group in which Piccolino was knocked down by RNAi (Regus-Leidig et al., 2014)—an approach with inherently variable and unknown knockdown efficiency—similarly reported SR collapse ([Müller et al., 2019]; this study) but no apparent tethering defect. A partial reduction of Piccolino may lead to SR collapse, but leave sufficient tethers intact to maintain near-normal SV density.

Further supporting a role of Piccolino as an SV tether at photoreceptor ribbons is the finding that SV density decreased selectively in the ribbon RP but not in the cytoplasmic RP upon loss of Piccolino (see Fig. 2). This selective reduction of SVs at the SR is important, given the findings of a recent study investigating the zebrafish lateral line organ, which also forms ribbon synapses (David et al., 2024). The study investigated SV pools following deletion of the motor protein Kif1aa. Motor proteins have previously been suggested to act as SV tethers (but see discussion below). Interestingly, the loss of Kif1aa led to a decrease in the number of SVs at both the ribbon RP and the cytoplasmic RP. This broader reduction suggests a secondary effect on the number of SVs in the ribbon RP, rather than a direct tethering defect, unlike the selective depletion of SVs in the ribbon RP upon loss of Piccolino.

### Topology of Piccolino at rod photoreceptor ribbon synapses implicates its N terminus in SV tethering

A key finding of our study is that Piccolino is oriented at rod photoreceptor ribbon synapses. This differs from a previous study that investigated the *in situ* topology of Piccolino at retinal ribbon synapses and found a regular topology of Piccolino (Limbach et al., 2011). Piccolino was similarly far away from the SR (∼ 30 nm), regardless of which epitope of Piccolino was targeted, N- or C-terminal (Limbach et al., 2011). The same study also found the protein CtBP2, which is usually restricted to the SR matrix (tom Dieck et al., 2005; Magupalli et al., 2008; Papadopoulos et al., 2024), at some distance from the SR matrix. The authors speculated that the long fixation time required for pre-embedding immuno-EM may have prevented the primary antibodies from penetrating into the SR matrix (Limbach et al., 2011), which could have obscured a potential orientation. Considering these findings, we revisited the nanoscale topology of Piccolino using short fixed mouse retinal preparations for STED and immuno-EM. Both super-resolution techniques clearly demonstrated that the C terminus of Piccolino is located within the SR matrix (see Fig. 3). This finding is consistent with a previous study showing an interaction between the C terminus of Piccolino and RIBEYE (Müller et al., 2019), which had already suggested a close association of Piccolino with the SR matrix. Thus, the localization of the C terminus within the SR matrix confirmed in this study provides additional data that explain the ribbon collapse upon loss of Piccolino in Pclo^cKO^ rod photoreceptors. In contrast, the N terminus of Piccolino is located ∼ 30 nm from the SR matrix. Notably, this distance closely matches the tether length in ribbon synapses in the frog and mouse retina (Usukura and Yamada, 1987; Graydon et al., 2014) and in IHCs of the mouse cochlea (Chakrabarti et al., 2018).

### Could ALPS-mediated protein–lipid binding mediate SV tethering?

Two concepts have been proposed to govern SV tethering to the SR and subsequent transport toward the AZ: the conveyor belt model, in which SVs undergo activity-dependent, i.e., motor protein-driven, attachment and transport along the SR, and the safety belt model, in which SVs attach and move passively along the SR before the SVs fuse with the plasma membrane at the base of SRs (Parsons and Sterling, 2003). The conveyor belt model has largely been ruled out based on observations that tethering and transport are likely to be ATP-independent (Heidelberger et al., 2002), and that the speed of transport would be too slow to account for the number of SVs released during stimulation (Parsons and Sterling, 2003). The safety belt model assumes a more passive role for the ribbon in transporting SVs and is supported by a number of studies showing that SR-tethered SVs are slowed down upon binding to the SR (Holt et al., 2004; LoGiudice et al., 2008; Babai et al., 2019). The velocity of the SV transport by lateral diffusion along the ribbon is sufficient to sustain tonic neurotransmitter release (Graydon et al., 2014; Joselevitch and Zenisek, 2020).

We have identified an ALPS motif at the N terminus of Piccolino that may contribute to SV tethering. There are several reasons why it fits the passive model of SV transport. First, the insertion of the Pclo ALPS motif (and ALPS motifs in general) into lipid bilayer defects is spontaneous (see Fig. 4 [Vanni et al., 2013]) and does not require ATP. Second, it has been suggested that SVs move along the SR by undergoing a series of successive binding and unbinding steps to and from tethers, a process termed “crowdsurfing” (Graydon et al., 2014). Crowdsurfing requires tethers that strike a delicate balance between strong enough binding of SVs to the SR, yet weak enough binding to allow unbinding for directional movement of SVs. Supporting this idea, an ALPS-like motif in complexin I, a protein that binds to the SNARE complex and modulates SV fusion, has been shown to exhibit relatively weak binding (K_D_ ∼ 10–20 µM; [Snead et al., 2014]). Weak individual protein–lipid interactions are likely stabilized through the avidity of multiple tethers rather than the affinity of a single tether, consistent with the observation that multiple tethers bind to individual SVs (Usukura and Yamada, 1987). Third, a potential tether should be able to bind its cargo with high fidelity. Our MD simulations and biochemistry data demonstrate a strong preference of the Pclo ALPS motif for liposomes that match SVs in composition and size (see Figs. 4 and 5). *In vivo*, such a high selectivity could enable the discrimination between actual SVs and other membranous organelles present in synaptic terminals, such as mitochondria, plasma membrane, or endosomes. At the Golgi apparatus, ALPS-mediated binding has already been shown to function as a filter that selects between different cargo vesicles (Magdeleine et al., 2016). Furthermore, the synaptic protein synapsin I has been shown to bind to SVs via an ALPS motif in activity-dependent cycles of SV association and dissociation, organizing the SV reserve pool in conventional chemical synapses (Krabben et al., 2011). However, it is important to note that the evidence presented here for ALPS-mediated SV tethering by Piccolino is indirect. Definitive proof would require, for example, the reintroduction of a membrane-binding–deficient ALPS mutant of Piccolino into Pclo^cKO^ rod photoreceptors. Moreover, we consider the possibility that ALPS-mediated tethering by Piccolino is not the sole mechanism anchoring SVs to the SR. Analogously, the Golgi tethering protein GMAP-210 captures vesicles via its ALPS motif, while Rab binding—albeit not required for vesicle binding *per se*—is additionally needed for stable *in vivo* tethering at the cis-Golgi (Sato et al., 2015), suggesting that a Rab GTPase may act alongside Piccolino at ribbon synapses. In this context, Rab3A is an attractive candidate, having previously been implicated in SV replenishment at ribbon synapses of *Ambystoma tigrinum* (Tian et al., 2012).

### Is Piccolino the only tether present at retinal ribbon synapses?

While we found some SVs associated with the SRs in the Pclo^cKO^ rod photoreceptors, these showed clearly different characteristics. They were located at a much shorter distance (<5 nm) to the SR compared with the ∼30 nm in Pclo^WT^ rod photoreceptor SRs (see Fig. 2, C, G, and K). This suggests that SRs retain some affinity to SVs in the absence of Piccolino. Previously, it was suggested that RIBEYE, the main component of SRs, binds to SVs by providing a sticky surface to which SVs can attach (Schwarz and Schmitz, 2017). However, it is unclear whether this direct binding can replace the function of SV tethers. Investigating whether direct binding of SVs to RIBEYE is important for ribbon synapse function is difficult because deletion of RIBEYE abolishes SRs, and thus tethered SVs altogether (Maxeiner et al., 2016). A simpler explanation for the presence of SVs on the Pclo^cKO^ rod photoreceptor SRs is that photoreceptor terminals are filled with thousands of highly mobile SVs (Holt et al., 2004; LoGiudice et al., 2008). This allows the SVs to come into close contact with the SRs, which does not occur in the presence of an SV tether acting as a molecular spacer. Importantly, the role of SV tethers is not to attach SVs to the SR, but to capture and position SVs at the correct distance for directed transport along the ribbon to the AZ. Therefore, close-contact association of SVs with the SR in the absence of tethers is unlikely to be physiologically relevant and cannot substitute for the tethering function of Piccolino.

Despite the significant reduction in tethered SVs in Pclo^cKO^ rod photoreceptor ribbon synapses, a small number of tethered SVs remains detectable. Two factors may account for this residual population. First, the Cre-driver used in this study becomes active around postnatal day 6 (Li et al., 2005), which is after the onset of Piccolino expression (detectable as early as postnatal day 2; [Regus-Leidig et al., 2009]). This leaves open the possibility that a small number of Piccolino copies were already incorporated into the SR prior to gene deletion, capable of forming tethers. Second, the higher accelerating voltage of the high-power TEM (200 kV) used for EM tomograms increases beam penetration and reduces image contrast at the ribbon border. This makes it difficult to unambiguously delineate the SR border from putative tethers in edge cases. Such ambiguous structures were conservatively classified as tethered SVs to minimize bias. Importantly, neither factor alters the core finding of the nearly complete loss of SV tethers in Pclo^cKO^ rod photoreceptor ribbon synapses. This strengthens the role of Piccolino as the primary tethering component at the rod photoreceptor ribbon synapse.

Taken together, the findings of this study demonstrate that Piccolino is the long-sought-after missing link that tethers SVs to the SRs in photoreceptor synapses.

## Materials and methods

### Animals

Mice (ms) were group housed and maintained on a 12/12-h light/dark cycle with light onset at 6:00 A.M. and with food and water *ad libitum*. Mouse breeding was performed in the Biotechnologisches Forschungslabor of the Friedrich-Alexander-University of Erlangen-Nürnberg according to European, German, and local guidelines for the welfare of experimental animals.

For preparation of primary cultures, E18 Sprague-Dawley rats (RjHan: SD) obtained from Janvier Labs were used. The experiments were carried out in accordance with the European Council Directive (2010/63/EU, amendment 2019) and local regulations. They were registered and approved by the local authorities Regierung Unterfranken (55.2.2-2532-2-1802). Attrition: No animals were excluded from the analysis.

Immuno-EM experiments examining the nanoscale organization of Piccolino were conducted on 2–3-mo-old male and female C56BL/6N animals. Conditional, rod photoreceptor–specific Piccolino knockout ms (hereafter named Pclo^cKO^ ms) used in this study were generated as follows: a targeting cassette for the generation of a “knock-out first” allele (tm1a; [Skarnes et al., 2011]) was inserted into exon 4 of the *Pclo* gene in embryonic stem cells generated by the European Conditional Mouse Mutagenesis Program. Embryonic stem cells harboring the Pclo^tm1a^ allele were inserted into superovulated C56BL/6N ms by blastocyst injection. A conditional allele was generated by crossbreeding Pclo^tm1a^ ms with Flippase-expressing ms (Tg(ACTFLPe)9205Dym; Strain #003800; Jackson Laboratories; [Rodríguez et al., 2000]), resulting in floxed Piccolo ms (Pclo^tm1c^) expressing WT Piccolo and Piccolino. Cell-specific knockout of the *Pclo* gene in rod photoreceptors was achieved by crossbreeding floxed Pclo^tm1c^ ms with ms expressing the codon-improved iCre recombinase under the rhodopsin promotor (B6.Cg-Pde6b + Tg(Rho-icre)1Ck/Boc; Strain #015850; Jackson Laboratories, Li et al., 2005). Floxed, Cre-negative littermates served as WT controls in all experiments (hereafter named Pclo^WT^).

### Genotyping

Genomic DNA for genotyping was extracted as described previously (Ryl et al., 2021). Briefly, ear or tail biopsies were treated with 5% Chelex 100 (Bio-Rad Laboratories) at 95°C while shaking gently. After brief centrifugation (13,000 × *g*), 3 μl of the supernatant served as PCR template. The following primers were used:

For iCre detection (650 bp):

FWD 5′→3′, RH1.1: 5′-TCA​GTG​CCT​GGA​GTT​GCG​CTG​TGG-3′

REV 5′→3′, iCre550: 5′-CTT​AAA​GGC​CAG​GGC​CTG​CTT​GGC-3′ (Li et al., 2005).

For pclo wt detection (511 bp):

FWD 5′→3′, PcloEx4afor: 5′-AAC​TGT​CAG​ACA​CAG​AGA​GCA​A-3′

REV 5′→3′, PcloEx4brev: 5′-TGC​CTA​TGG​GTC​CTT​CTT​CG-3′.

For detection of floxed pclo alleles (645 bp):

FWD 5′→3′, PcloEx4afor: 5′-AAC​TGT​CAG​ACA​CAG​AGA​GCA​A-3′

REV 5′→3′, PcloIfitm2: 5′-CCC​CTC​TTT​TCA​CAC​ACA​C-3′.

### Antibodies

Primary antibodies raised in rabbits (rb), guinea pigs (gp), ms, and goats (gt) and suitable secondary antibodies were used for immuno-EM, immunocytochemistry (ICC) or WB. Primary antibodies: AZ: gp anti-BsnEx1 (custom antibody raised against Exon 1 of Bsn, Davids Biotechnologie GmbH), rb anti-Ca_v_1.4 (ICC: 1:2,000, cat. no. 365003; SySy), rb anti-ubMunc13-2 (batch 52; ICC 1:6,000, [Cooper et al., 2012]), rb anti-RIM1ab1 (detects RIM2α, see [Löhner et al., 2017], cat.no. 143003; SySy) SR: ms anti-CtBP1 (ICC: 1:1,000, cat. no. 612042; BD Transduction Laboratories), rb anti-Pclo4 (EM 1:1,000; ICC 1:3,000 [Spiwoks-Becker et al., 2008]), gp anti-Pclo4 (ICC 1:5,000, WB 1:1,000, a kind gift of A. Fejtová, Universitätsklinikum Erlangen, Erlangen, Germany), rb anti-Pclo (ICC 1:10,000, cat.no. 142113; SySy), rb anti-Pclo49 (EM, 1:500; ICC 1:1,000, custom antibody raised against the last 23 AAs of Piccolino, Biotrend, [Regus-Leidig et al., 2013]), rb anti-Pclo ALPS (WB 1:1,000, custom antibody raised against the ALPS motif of Piccolo/Piccolino, immunogen AAs V^865^-L^883^, Biotrend), gp anti-RIBEYE A-Domain (ICC 1:10000, cat. no. 192104; SySy). Cell markers: ms anti-Brn3A (ICC 1:1,000cat.no. MAB1585; MilliporeSigma), gp anti-Calbindin (ICC: 1:2,000, cat. no. 214005; SySy), rb anti-Cone Arrestin (ICC: 1:3,000, cat. no. AB15282; MilliporeSigma), rb anti-GABA (ICC: 1:2,000, cat. no. A0310; MilliporeSigma) gt anti-GlyT1 (ICC, 1:10,000, cat. no. AB 1770; Chemicon, Temecula), GABA, ms anti-PKCα (ICC: 1:20,000, cat. no. 610107; BD Transduction Laboratories), rb anti-Secretagogin (ICC: 1:10,000, cat.no. BVD-RD181120100-0.1; BioVendor). Other: gp anti-vGLUT1 (ICC, 1:1,000, cat.no. AB5905; Chemicon); ms anti-Grp75 (ICC 1:400, cat.no. ab2799; Abcam), ms anti-strep Tag (WB, 1:5,000, cat. no. SAB2702216; MilliporeSigma). Secondary antibodies: suitable Alexa 488 (anti-ms, rb, gp), Alexa 555 (ms, rb, gp) and Alexa 647 (ms, rb, gp, gt) dyes (Thermo Fisher Scientific) were used for standard immunofluorescence stainings. STED antibodies: STAR Orange gt anti gp and STAR RED gt anti rb (ICC 1:200, cat. nos. STORANGE-1001, STRED-1001; Abberior Instruments). Western blot/dot blot antibodies: HRP-coupled gt anti rb (WB 1:10,000, cat. no. 7074S; Cell Signaling) and gt anti-ms (Biozol, WB 1:10,000, cat. no. BLD-405306). Immuno-EM antibodies: Fluoronanogold (FNG) antibodies: Alexa Fluor 488 - FNG Fab’ gt anti rb (EM 1:100, cat. no. 7003; Nanoprobes Inc.).

### Retina preparation

Preparation of retinal tissue and antibody incubation for immunofluorescence staining was performed as described previously with minor modifications (Müller et al., 2019). ms were anesthetized by inhalation of isoflurane and sacrificed by cervical dislocation. Lens and vitreous body were removed from the eyecup, and retinae were fixed for 15 min in 4% PFA in PB buffer (0.1 mM) before mounting for cryosectioning in Tissue-Tek O.C.T. (CLSM; Sakura FineTek) or mounting with solid antifade (STED; cat.no. MM-2013;Abberior Instruments). Retinae were cut into 14-µm-thick vertical sections with a cryostat (CM3050 S, Leica Microsystems).

### Primary rat cortical cultures

Primary cortical cultures from rat E18 embryos were prepared as described previously (Anni et al., 2021). Briefly, pregnant rats were anesthetized with isoflurane inhalation and decapitated using a guillotine. The embryos were rapidly isolated and decapitated, and brains were freed from skull and meninges in ice-cold HBSS medium (cat.no. 14175053). Cortices were isolated, pooled and incubated with 0.25% trypsin (cat.no. 15400054, both Thermo Fisher Scientific) at 37°C for 20 min. Tissues were then triturated using syringes with injection needles 0.90 and 0.45 mm in diameter in a solution of 0.1 mg/ml DNase I (cat.no. 11284932001; Roche). The resulting cell suspension was filtered through a 100-μm nylon cell strainer. Cells were seeded in DMEM (cat.no. 41966029; Thermo Fisher Scientific) containing 10% fetal calf serum (FCS, cat. no. S0015; Biochrom), 2 mM L-Glutamine (cat.no. 25030024), and 1% antibiotic/antimycotic (cat.no. 15240062, both Thermo Fisher Scientific) at a density of 200,000 cells in 1 ml per coverslip (12 mm Menzel glass in 24-well plates coated with 0.5 mg/ml poly-L-lysine, cat.no. P1524; MilliporeSigma). After 1 h, allowing cells to attach, the medium was replaced with Neurobasal Medium (cat.no. 12348017) supplemented with 2% B27 (cat. no. 17504044), 0.8 mM GlutaMAX (cat.no. 35050-038) and 1% antibiotic/antimycotic (all from Thermo Fisher Scientific). Cultures were maintained in a 5% CO_2_-containing humidified incubator at 37°C. Cells were fed with fresh Neurobasal medium once a week until infection.

### ICC and light microscopy

For ICC, retina cryosections or primary rat cortical neurons were blocked in blocking solution (10% normal gt serum [NGS], 1% bovine serum albumin [BSA], and 0.5% Triton-X-100 in 0.01 M PBS) for 1 h at RT. Primary antibodies were diluted in antibody dilution solution (3% NGS, 1% BSA, and 0.5% Triton-X-100 in 0.01 M PBS). Incubation was performed overnight at 4°C (CLSM) or for 4 days at 4°C (STED), secondary antibody incubation for 2 h at RT.

For analysis, labeled specimens were imaged at RT using the following microscopes. Overview images were taken using an Axio Imager M2 equipped with an ApoTome.2 (Carl Zeiss Microscopy GmbH) with a 20 × (0.8 NA, Plan Apochromat) or 40× objective (0.95 NA water, Plan Apochromat [Carl Zeiss AG]). Detail scans were imaged at an LSM 710 laser scanning microscope (Carl Zeiss Microscopy GmbH) with a 63× objective (1.4 NA oil, Plan Apochromat) as stacks of multiple optical sections. STED microscopy was performed at an Abberior Facility Line STED microscope (Abberior Instruments) at the Optical Imaging Center Erlangen with an Olympus 60 × objective (NA 1.42, oil). For 2-color STED, samples were excited with 561 and 640 nm and depleted with one pulsed STED laser at 775 nm in a line-interleaved fashion. Samples were imaged with a 25 nm pixel size. Maximum intensity projections of image stacks were created with Fiji ImageJ (https://imagej.net). Images were adjusted for contrast and brightness using Fiji ImageJ. Line profiles and denoising were performed using built-in tools of the Lightbox software (Abberior Instruments). Images were further deconvolved in Lightbox or processed with the TRUESHARP online deconvolution tool by Abberior (https://app.truesharp.rocks/). FWHM values were obtained by fitting line profiles with either a single or a double Gaussian function.

### EM

#### Fixation for good tissue preservation (“best fix”)

Retinae were treated as described previously (Gierke et al., 2020). Briefly, after removing cornea and lens, retinae were sequentially fixed in 4% PFA for 1 h and in 2.5% glutaraldehyde in 0.1 mM PB buffer for 2 h at RT, followed by postfixation in 2% osmium tetroxide and 1.5% potassium hexacyanidoferrate (II) in 0.1 M cacodylate buffer for 1.5 h. After dehydration in rising ethanol concentrations (30–100%) and propylene oxide, retinae were embedded in Epon resin (Fluka, Buchs, Switzerland).

#### HPF and FS

For HPF, retinae were treated as described previously with modifications (Möbius et al., 2010). Briefly, retinae were dissected in ice-cold, oxygenated Ames’ Medium. After removing the cornea and lens, four incisions were made in the eyecup and retina. The eyecup was gently flattened using brushes. A piece of nitrocellulose was used to pick up the flattened eyecup and turn it upside down. The eyecup was now gently removed from the retina with a pair of forceps. The retina was immediately cut vertically into 150-µm-thick sections using a Tissue Slicer (Stoelting Co.). The remaining procedure was performed as described previously with modifications (Jung et al., 2015). Individual sections were mounted into type A specimen carriers (Leica Microsystems, Wetzlar, Germany; 3 mm diameter, 0.2 mm depth) with a Perfect Loop (Science Services, Germany). The flat side of a type B carrier (Leica Microsystems, 3 mm diameter, 0.3 mm depth) was coated in 1-hexadecene and placed onto the sample containing a type A carrier. Assembled carriers were loaded into the HPF sample holder, and excess liquid was removed with filter paper. The sample holder was loaded into the HPM100 high-pressure freezer (Leica Microsystems) for cryofixation and storaged in liquid nitrogen. Next, high-pressure frozen samples were freeze-substituted using the EM AFS2 (Leica Microsystems) machine. In brief, specimens were incubated in 0.1% (wt/vol) tannic acid in acetone at −90°C for 4 days, followed by three washing steps in acetone at −90°C for 1 h, respectively. Next, 2% (wt/vol) osmium tetroxide in acetone was applied and incubated at −90°C for 7 h, before it was increased to −20°C (5°C/h). Next, the temperature was gradually increased to 4°C (10°C/h), and samples were washed in acetone three times (1 h each) and brought to RT. Finally, samples were infiltrated and embedded in epoxy resin (Glycidyl ether 100/EPON812, SERVA Electrophoresis GmbH); epoxy/acetone 1:1, 1 h; 100% epoxy resin overnight and 3–6 h on the next day. Polymerization was performed for at least 48 h at 70°C.

To assess the quality of the cryo-fixed tissues and to choose the region of interest, 2D electron micrographs were taken from 55-nm ultrathin sections at 60 kV using a Zeiss EM10 transmission electron microscope (Carl Zeiss) equipped with a Gatan Orius SC1000 camera (Gatan). After pre-screening, 200-nm semi-thin sections were cut and labeled on both sides of the poststained grids with 10-nm gold beads (Ted Pella Inc., Redding, CA, USA) as fiducial markers. Single-axis tomograms were acquired from −60° to +60° with 1° increments at 15,000 × magnification and a pixel size of 0.664 nm using a JEM2100Plus (JEOL) transmission electron microscope operated at 200 kV equipped with a 20 MP XAROSA bottom-mount CMOS TEM camera (EMSIS) and the Serial-EM software (Mastronarde, 2005). Tomograms were generated using the software package eTomo. 3D Reconstructions were generated using 3dmod (Kremer et al., 1996).

#### Pre-embedding immuno-EM

For pre-embedding immuno-EM, retinae were prefixed in 4% PFA in 0.01 mM PBS (pH 7.4) for 15 min (Pclo49 antibody) or 15–30 min (Pclo4 antibody) at RT and further processed as described previously albeit with modifications (Gierke et al., 2020). Briefly, after four freeze and thaw cycles in liquid nitrogen, retinae were embedded in buffered 3% low-melting agarose. Agar blocks were cut vertically into 60-μm sections with a vibratome (VT100S, Leica Microsystems). Sections were blocked in 10% NGS and 1% BSA in 0.01 mM PBS for 2 h, followed by incubation with primary antibodies for 4 days at 4°C. PBS-washed sections were incubated with secondary, FNG-coupled secondary antibodies. Nanogold particles were silver-enhanced using the HQ Silver Enhancement Kit (Nanoprobes) for 10 min in darkness. Silver-enhanced sections were protected from osmium tetraoxide treatment by gold toning in 0.05% chloroauric acid (5 min) and stabilized in 1% thiosulfate (5 min). After several washing steps in ddH_2_O, sections were postfixed in 2.5% glutaraldehyde in 0.1 mM cacodylate buffer. Specimens were further treated with 0.5% osmium tetraoxide in 0.1 M cacodylate buffer (30 min). Finally, samples were dehydrated in a rising ethanol series and flat-mounted in epoxy resin. For analysis, ultrathin sections were cut with an Ultracut E microtome (Reichert-Jung) and contrasted with lead citrate (Ultrostain 2, Leica Microsystems) and Uranyless (Delta Microscopies, Mauressac, France) in an automatic contrasting system (EM AC20, Leica Microsystems). 60-nm ultrathin sections were examined and photographed with an EM10 electron microscope operating at 60 kV (Carl Zeiss AG) equipped with a SC1000 Orius CCD camera (Gatan, Pleasanton, CA, USA) in combination with the DigitalMicrograph 3.1 software (Gatan).

### Vector generation

The Piccolo/Piccolino [Pclo-201: ENSMUST00000030691] construct Pclo mp2.1 (bp 2199–2961), which contains the ALPS motif, was amplified from a larger DNA construct (mp2, bp 1,221–3,945) generated in a previous study (Müller et al., 2019) by using the following primers:

FWD 5′→3′: 5-′ATG​GGA​GGT​GAA​CTG​GCA​GC-3′

REV 5′→3′: TGG​CTT​CTG​GGC​GAG​GAC-3′.

The deletion construct mp2.1^ΔALPS^ was generated by inverse deletion PCR using the mp2.1 construct as template and the following primers containing HindIII restriction sites:

FWD 5′→3′: 5-′TAT​AAG​CTT​AAC​TTA​ATT​TCC​ACA​GCA​GGT-3′

REV 5′→3′: 5-′TAT​AAG​CTT​AGT​TTC​TTG​AGG​AGT​TGT​GGG-3′.

After PCR, the non-mutated template was digested with DpnI. The amplicons were digested with HindIII and religated with T4 ligase. All constructs were subcloned into the pCR8/GW/TOPO vector via TA cloning (Gateway system, Life Technologies). The strep tag II was added to the 3′ end of mp2.1 and mp2.1^ΔALPS^ constructs via PCR using the following primers:

FWD 5′→3′: 5-′ATG​GGA​GGT​GAA​CTG​GCA​GC-3′

REV 5′→3′: 5-′TTA​TGC​AGA​TTT​TTC​AAA​TTG​TGG​ATG​AGA​CCA​AAC​TCC​AGA​AGG​CTT​CTG​GGC​GAG​GAC​G-3′.

For expression in primary cortical neurons, an N-terminal mCherry and C-terminal mitoTag were added to the mp2.1 and mp2.1^ΔALPS^ constructs using the Gateway recombination system in two sequential steps. First, N-terminal mCherry was introduced by L/R recombination of the pCR8/GW/TOPO mp2.1 or pCR8/GW/TOPO mp2.1^ΔALPS^ entry vectors with a Gateway destination vector encoding mCherry at the N terminus (pDest mCherry), yielding mCherry-mp2.1 and mCherry-mp2.1^ΔALPS^ constructs. Second, to add the C-terminal mitoTag, the mCherry-mp2.1 and mCherry-mp2.1^ΔALPS^ inserts were re-amplified by PCR, re-subcloned into pCR8/GW/TOPO entry vectors, and subsequently transferred by L/R recombination into a destination vector encoding the mitoTag at the C terminus, yielding the final mCherry-mp2.1-mitoTag and mCherry-mp2.1^ΔALPS^-mitoTag constructs. Finally, constructs were amplified by PCR using primers containing HindIII and NheI restriction sites, digested with HindIII and NheI, and ligated into the pAAV-hSynapsin vector using standard T4 ligation protocols.

For expression in bacterial cells, constructs were first amplified via PCR with primers encoding the strep-tag II and NheI/XhoI restriction sites.

FWD 5′→3′: 5′-ATA​CAT​GCT​AGC​ATG​ACA​ACG​GCC​CCT​CCT​TTG-3′

REV 5′→3′: 5′-GTG​GTG​CTC​GAG​TTA​TTA​TGC​ACT​CTT​TTC-3′.

Constructs were then inserted into the pET21(+) vector via NheI/XhoI restriction sites using standard T4 ligation protocols. Correct bp sequences of all plasmids were confirmed by Sanger Sequencing (Eurofins Genomics) before use.

### AAV production and infection

AAV particles were generated by co-transfection of pAAV-hSynapsin::mCherry::mp2.1::mitoTag or pAAV-hSynapsin::mCherry::mp2.1 ^ΔALPS^:: mitoTag together with the AAV packaging plasmid pAAV-DJ and the helper plasmid pAAV-Helper into HEK293T cells. Three days after transfection, cells were collected and lysed by three freeze-and-thaw cycles using liquid nitrogen. Subsequently, cell lysates and supernatants were cleared by sequential centrifugation (750 × *g*, then 2,000 × *g*, 4°C, 10 min each) to remove cells and residual debris. Viral particles were precipitated by overnight incubation in PEG/NaCl solution at 4°C, followed by centrifugation (2,500 × *g*, 4°C, 1 h). The supernatant was discarded, and the pellet was resuspended in TNE buffer (50 mM Tris-HCl, 100 mM NaCl, and 0.1 mM EDTA). Purified viral particles were aliquoted and stored at −80°C until use. For infection, 1–3 μl of the AAVs were added to DIV14 cortical neurons. Seven days post infection, cells were fixed using 4% PFA and processed for ICC.

### Protein overexpression and purification

Plasmids encoding strep-tagged Piccolino constructs were transformed into BL21 by standard heat shock protocols and plated on LB_amp_. Single colonies were inoculated in 5 ml LB_amp_ as overnight culture. The next day, the overnight culture was mixed with 450 ml fresh LB_amp_ to a starting OD_600_ of 0.05. Growth was monitored until OD_600_ >0.75, when overexpression was induced by addition of 1 mM IPTG. After 4 h at 30°C, cells were harvested by centrifugation. Pellets were resuspended and incubated for 30 min in ice-cold lysis buffer (0.1 M Tris-HCl, 0.15 M NaCl, and 1 mM EDTA, pH 8.0). Protease inhibitor cocktail (cat. no. 04693116001; Roche Diagnostics), 10 mg/ml lysozyme, and 3 U/ml benzonase were added freshly prior to use. Cells were disrupted by 10 cycles á 10 s with a tip sonicator (50% intensity, Bandelin Electronic GmbH, Berlin, Germany) with cooling in between rounds. Next, samples were centrifuged (30 min, 4°C, 20,000 × *g*) to pellet insoluble material. Supernatants of cleared lysates were diluted 1:1 in Buffer W (IBA Lifesciences, 0.1 M Tris 0.15 M NaCl, and 1 mM EDTA, pH 8.0) and loaded onto prepacked Strep-Tactin Superflow columns (cat. no. 2-1209-001; IBA Lifesciences) and allowed to flow through by gravity. After binding, columns were washed with five column volumes of Buffer W. Strep-tagged proteins were displaced from the columns by addition of Buffer E (IBA Lifesciences, 0.1 M Tris/HCl pH 8.0, 0.15 M NaCl, 1 mM EDTA, and 2.5 mM desthiobiotin). The obtained eluates were placed onto Amicon centrifugal filter units with 10 kDA MWCO (cat.no. UFC201024; Merck Millipore) to exchange the elution buffer with HKM Buffer (50 mM HEPES, 120 mM potassium acetate, and 1 mM MgCl_2_, pH 7.4).

### Liposome preparation and size determination

All phospholipids (PLs) used in this study were purchased dissolved in chloroform from Avanti Polar Lipids. Preparation of artificial liposomes was performed according to the “Morrissey Lab Protocol for Preparing PL Vesicles (small unilamellar vesicle [SUV]) by Sonication” (https://tf7.org/suv.pdf) with modifications. Briefly, 2 μmol total PLs were dispensed in a glass tube. To generate SUVs of an SV-like composition, DOPC (cat.no. 850375), DOPS (cat.no. 840035), DOPE or DOPE-NBD (1,2-dioleoyl-sn-glycero-3-phosphoethanolamine-N-[7-nitro-2-1,3-benzoxadiazol-4-yl], cat. no. 810145), and CHOL (50:9:20:1:20 molar ratio) in chloroform were mixed and dried down under a gentle stream of nitrogen. When dry, the lipid film was placed in a rotary evaporator for 45 min to remove residual chloroform. To the dried-down PL mix, 2 ml HK buffer (50 mM HEPES and 120 mM potassium acetate, pH 7.4) was added and incubated for 1 h at RT with occasional mixing. The rehydrated suspension was then vortexed vigorously to obtain a solution of multi-lamellar vesicles. SUVs were generated by extrusion of the rehydrated lipids through polycarbonate filters containing the desired pore sizes (200, 100, 50, 30 nm) 21 times. SUVs smaller than 30 nm diameter were generated by sonication with a tip sonicator for 20 min (20% power, 6 s on/4 s off intervals) and cooling in between to prevent overheating. The sonicated lipid mixture was additionally extruded through a 30-nm filter to exclude bigger liposomes from the mixture. The final product is a 1 mM PL SUV solution in HK buffer. Liposome size was determined via DLS. Samples were measured at 173° backscatter at 20°C in a Zetasizer Nano ZS (Malvern Panalytical GmbH). Each mixture was measured three times á 15 runs. For use, liposome mixtures had to meet the predefined, software-generated quality indicators (correlation function intercept, baseline accuracy, acceptable count rate, and PdI limits).

### Liposome flotation assay

To investigate protein–lipid interactions, flotation assays were performed as follows:

Equal volumes of PLs (0.5–1 mM) and recombinant protein or peptide (5–10 μM) were mixed and incubated for 15 min at RT to allow for potential binding. For the competition experiment, purified protein was additionally incubated at a 1:1 molar ratio with anti-ALPS antibody for 30 min prior to mixing with SV-like liposomes. The mixture was adjusted to 50% sucrose (in HKM buffer) and served as the bottom fraction of a step gradient, which was overlaid with two steps containing 40% sucrose in HKM buffer (middle fraction) and HKM buffer without sucrose (top fraction). After ultracentrifugation (Optima-Max-XP, MLS-50 swing bucket rotor, both Beckman Coulter at 250,000 × *g*, 2.5 h, 20°C), successful migration of lipids to the top fraction was monitored by NBD fluorescence on a blue/green LED trans illuminator (Nippon Genetics). Fractions were carefully collected with a Hamilton syringe from bottom to top. Presence of protein in the collected fraction was determined by dot blotting.

### Dot blotting

Proteins were detected by spotting equal volumes (5–10 μl) per sample onto a nitrocellulose membrane and dried completely. Membranes were blocked in 5% skimmed milk powder in TBS-T buffer and incubated with primary anti-strep antibodies in 1% skimmed milk powder in TBS-T buffer. Secondary, HRP-coupled antibodies were diluted in TBS-T. After extensive washing, HRP-conjugated secondary antibodies were visualized by chemiluminescence detection (Luminata Forte, Merck Millipore). Lipids were visualized by NBD fluorescence. Images were obtained with a molecular imager (ChemiDoc XRS, Bio-Rad Laboratories).

### WB

Proteins were separated by SDS-PAGE using self-cast 10% Tris-Glycine-SDS gels. For detection of large proteins (i.e., Piccolo/Piccolino), proteins were separated on precast nuPAGE 3–8% Tris-acetate gels (cat. no. EA0378BOX; MilliporeSigma). For visualization of total protein, 10 μl of 2,2,2-TCE (cat. no. T54801; MilliporeSigma) was added to the gel mixture before polymerization. Proteins were transferred to PVDF membranes (Immobilon-P, cat. no. IPVH00010; MilliporeSigma) by semidry blotting (40 min, 17 V, RT, Trans-Blot Cell, Bio-Rad Laboratories) or tank blotting (3 h, 0.2 A, 4°C). For immunodetection, membranes were blocked with 5% skimmed milk powder in TBS-T before the application of primary antibodies. HRP-conjugated or fluorophore-conjugated secondary antibodies were diluted in 1% skimmed milk powder in TBS-T. Proteins were visualized by chemiluminescence detection (Luminata Forte, MilliporeSigma). Images were obtained with a ChemiDoc XRS (Bio-Rad Laboratories). Fluorescence blots were imaged in an iBright FL1500 gel documentation system (Thermo Fisher Scientific). Images were adjusted for contrast and brightness using CorelDRAW X9 (Corel Corporation).

### ALPS motif: structure prediction and visualization

For visualization of the ALPS motif in 2D, the predicted AA sequence was imported into the Heliquest software (Gautier et al., 2008). A vector graphics version of the result was created with CorelDRAW 2024. To predict whether the ALPS motif indeed forms an α-helix, the secondary structure of the AA sequence corresponding to the mp2.1 fragment used for lipid-binding studies was predicted using ColabFold, an optimized version of AlphaFold2 (Mirdita et al., 2022). Visualization of the predicted α-helical ALPS motif in 3D was performed using ChimeraX (Pettersen et al., 2021).

### MD simulations

All-atom and CG MD simulations were performed to investigate the binding dynamics and curvature-inducing properties of the Piccolino ALPS motif. Two membrane compositions were examined: (1) a pure DMPC membrane, and (2) a SV-like membrane (Takamori et al., 2006) enriched in CHOL, composed of 40% CHOL, 25% DOPE, 22% POPC, 8% DOPS, and 5% SM18. The Piccolino sequence used corresponds to the predicted ALPS motif (AAs V^873^-T^894^, X-VTGKLFGFGASIFSQASNLIST-X) (Drin et al., 2007).

#### ALPS membrane interaction: All-atom MD simulations

Binding behavior of the ALPS motif to lipids was investigated with 1-µs-long all-atom MD simulations. Systems were solvated explicitly and charge neutralized via the addition of counterions. Initial CG systems were constructed and equilibrated using DAFT (Pluhackova et al., 2015; Pöhnl et al., 2023a), with lipid bilayers generated via the *insane* tool (Wassenaar et al., 2013). The ALPS peptide was initially placed 5–6 nm above the bilayer surface. After energy minimization (500 steps), systems were equilibrated for 10 ps in the NVT ensemble with positional restraints (1,000 kJ mol^–1^ nm^–2^) applied to the peptide backbone, followed by 20 ns of NPT ensemble equilibration (Wassenaar et al., 2013) using the polarizable Martini force field (Yesylevskyy et al., 2010). Atomistic conversion was performed using initram (Wassenaar et al., 2014), and the ALPS motif was modeled as an α-helix with acetylated N- and amidated C-termini. All simulations were executed in GROMACS v. 5.0.5 (Pronk et al., 2013; Pall et al., 2015). Using the AMBER14 force field (Case et al., 2014) with Slipids parameters for lipids (Jämbeck and Lyubartsev, 2012a; Jämbeck and Lyubartsev, 2012b; Jämbeck and Lyubartsev, 2013). The simulation protocol followed (Pluhackova et al., 2015), with the exception that temperature coupling was achieved using the v-rescale thermostat (Bussi et al., 2007) set to 310 K. Hydrophobic defects were analyzed using the GROMACS *sasa* tool.

#### ALPS-induced membrane curvature: CG MD simulations

To assess the curvature-inducing capacity of the ALPS motif, we employed bicelle systems following the protocol by (Pöhnl et al., 2023a) to study unbiased protein-induced membrane curvature by employing distinct rim and core lipid compositions and cylindrical flat-bottomed potentials, while maintaining the native properties of infinite membrane systems (Pöhnl et al., 2023a; Pöhnl et al., 2023b). Simulations were conducted at CG resolution using the Martini2.2 force field (Marrink et al., 2004; Marrink et al., 2007; Monticelli et al., 2008; de Jong et al., 2013).

Four bicelle systems were generated with an approximate bicelle radius of 13 nm: two with an SV-like composition and two with DMPC cores. Each bicelle rim contained ∼450 DTPC and DOPC lipids, solvated by ≈140,000 water molecules with a 0.15 NaCl solution and counter ions. Cylindrical flat-bottomed potentials (500 kJ mol^–1^ nm^–2^) were applied. For SV-like domains, the outer and inner cylindrical radii were set to R_outer_ = 11.0 nm and R_inner_ = 8.5 nm (inverted potential [Pöhnl et al., 2023a]), respectively. For DMPC cores, these values were R_outer_ = 10.1 nm and R_inner_ = 7.7 nm. The simulations were conducted at 320 K using the *Verlet* integration scheme with conservative Martini parameters for neighbor lists (Kim et al., 2023). Electrostatics were treated with the Particle-Mesh-Ewald method (dielectric constant = 15 [Darden et al., 1993]), and bond constraints were applied using LINCS with parameters optimized for the improved CHOL model (Fábián et al., 2023). Following a 1 µs equilibration, one ALPS motif was positioned 1 nm above the bicelle surface with hydrophobic residues facing the membrane to promote interaction. The peptide was CG using Martinize2 with elastic bonds (Kroon, 2020) and restrained laterally using a cylindrical flat-bottom potential (R_outer_ = 5.0 nm) to avoid rim interactions. Termini were neutralized. Each system was simulated for 10–20 µs using GROMACS 2024.4 (Abraham et al., 2015; Páll et al., 2020). Membrane curvature was analyzed following the method by (Pöhnl et al., 2023a). Errors were estimated using block averaging.

### Quantification

#### Topology of Piccolino

For the distance analysis of FNG-positive puncta, each ribbon was labeled by a line through the center in ImageJ with the straight line equation

y=m*x+b

Subsequently, each FNG-positive point was marked via the MultiPoint tool, and the coordinates were exported via the ROI manager. The shortest distance (d) between a point and a line was then calculated by the equation

$$d=\frac{\left(m_{X1}+b_{Y1}+c\right)}{\sqrt{m^{2}+b^{2}}}$$

#### SV analysis

For the analysis of SV concentration in the cytoplasmic reserve pool, SVs in EM micrographs that were further away from AZs or postsynaptic elements were surrounded with the free-hand tool (ImageJ), counted, and divided by the area to receive SVs/µm^2^. For the analysis of SVs density in the ribbon RP, the surface of SRs was marked with a free-hand line. Next, SVs within 50 nm of the SR were counted and divided by the SR length to obtain the density of SVs/µm. The first 100 nm of SRs perpendicular to the AZ were excluded from the analysis to eliminate docked SVs from the quantification. For the analysis of the SV distance, the shortest distance between an SV and the border of the SR was determined in ImageJ using the straight line tool.

#### SV tethering analysis

For the identification of tethered SVs in EM tomograms, SVs were classified as tethered if a continuous, electron-dense physical link was visible between the SV membrane and the surface of the SR. Tethers were identified by visual inspection in individual tomographic virtual sections. The criterion for tethering was the presence of such a structural connection, not SV proximity to the ribbon *per se*. SVs located within 50 nm of the SR but lacking a visible link were scored as non-tethered. Accordingly, they were assigned to one of two categories: tethered (+ tether) or non-tethered (− tether). The fraction of tethered and non-tethered SVs was calculated per tomogram and compared between Pclo^WT^ and Pclo^cKO^ rod photoreceptors.

#### SV recruitment analysis

To quantify mitochondrial targeting of mCherry-tagged constructs and recruitment of SVs to mitochondria, infected neurons immunolabeled for Grp75 and vGLUT1 were imaged by confocal laser scanning microscopy as described above. The Grp75 channel was used to generate a binary mask of mitochondrial ROIs using Otsu’s thresholding algorithm and the “Make Binary” function in Fiji ImageJ. Mean fluorescence intensities of the mCherry and vGLUT1 channels were measured within each Grp75-defined ROI. Pearson’s *r* was calculated for each ROI between (1) Grp75 and mCherry signal intensities as a measure of construct targeting efficiency to mitochondria, and (2) Grp75 and vGLUT1 signal intensities as a measure of SV recruitment to mitochondria.

### Statistics

All statistical analysis and graph generation were performed using GraphPad Prism 10 (GraphPad Software Inc., San Diego, CA, USA). For statistical analysis, we calculated mean values from individual data points for each animal or experiment, respectively. The calculated mean values for each animal group or each individual experiment were compared with each other either using Student’s *t* test (two groups), or one-way ANOVA (>2 groups). Corrections for multiple comparisons or non-normally distributed data were applied wherever necessary (indicated in the respective figure legends).

### Online supplemental material

Fig. S1 shows PclocKO retinae exhibit normal retinal anatomy. Fig. S2 Shows localization of SR and AZ proteins in PcloWT and PclocKO retinae. Fig. S3 Shows binding of the Piccolino ALPS motif to SV-like liposomes and endogenous SVs.

## Supplementary Material

Review History

SourceData F1 is the source file for Fig. 1.

SourceData F5 is the source file for Fig. 5.

SourceData FS3 is the source file for Fig. S3.

## Data Availability

The uncropped Blots displayed in Figs. 1, 5 and S3 are available in the data. The numerical data underlying each figure are openly available in Zenodo (accession number: https://doi.org/10.5281/zenodo.20762753). Large imaging datasets are available from the corresponding author upon reasonable request.
