# Supplementary material for: The missing link: Piccolino is essential for tethering synaptic vesicles to rod photoreceptor ribbons
Source: J Cell Biol. 2026 Jul 23;225(9):e202509110. doi: 10.1083/jcb.202509110 (PMC13394108; doi:10.1083/jcb.202509110)
Supplement: SourceData F1 — is the source file for Fig. 1. [file jcb_202509110_sourcedataf1.pdf]

Fig1 E

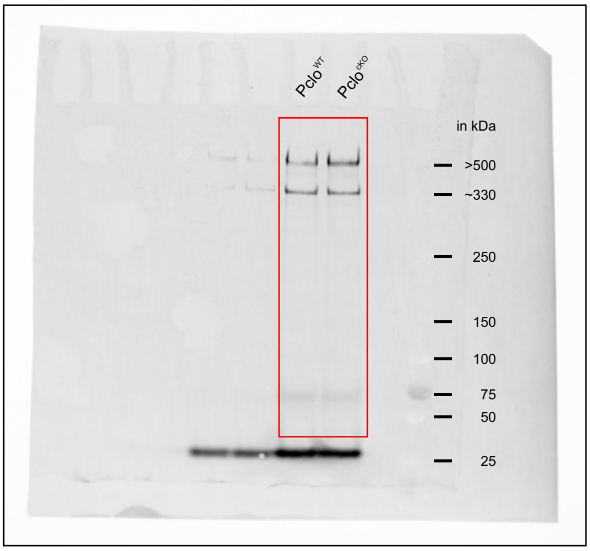

Fluorescence Blot, grayscale

Primary antibodies:
Pclo4 (rb)
Synaptophysin (rb)
Secondary antibodies:
anti-rb AlexaFluor800 (green)
anti-rb AlexaFluor555(red)

Synaptophysin = blotting control

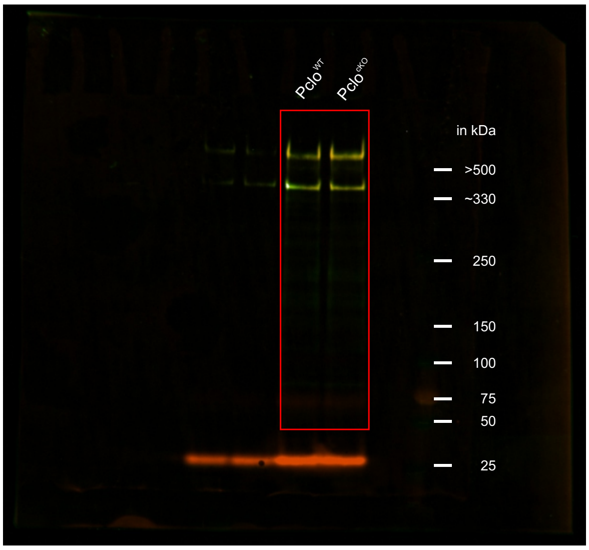

Fluorescence Blot, color

Primary antibodies:
Pclo4 (rb)
Synaptophysin (rb)
Secondary antibodies:
anti-rb AlexaFluor800 (green)
anti-rb AlexaFluor555(red)

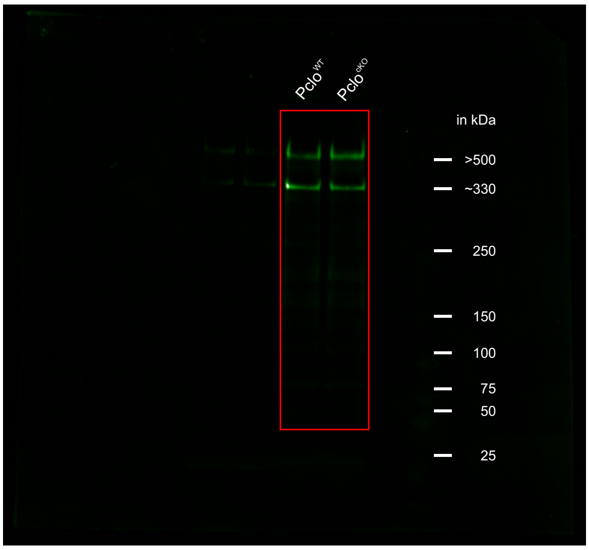

Fluorescence Blot, color

Primary antibodies:
Pclo4 (rb)
Secondary antibodies:
anti-rb AlexaFluor800 (green)
