# Supplementary figures and images for: The missing link: Piccolino is essential for tethering synaptic vesicles to rod photoreceptor ribbons
Source: J Cell Biol. 2026 Jul 23;225(9):e202509110. doi: 10.1083/jcb.202509110 (PMC13394108; doi:10.1083/jcb.202509110)

**Fig5 B**

TCE-stained SDS PAGE

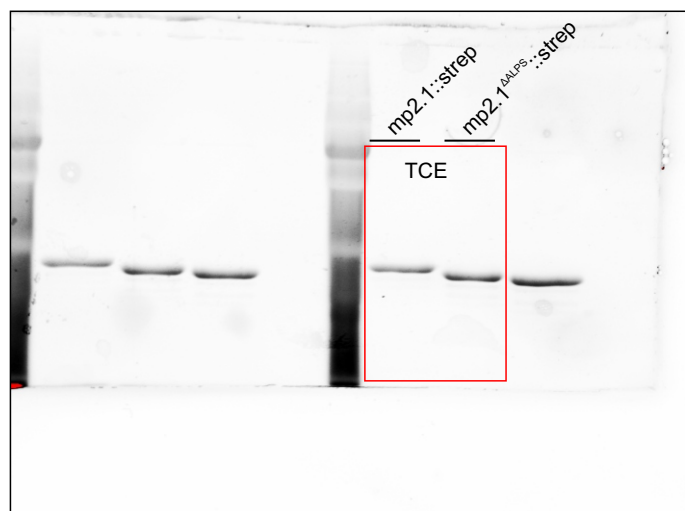

Western Blot

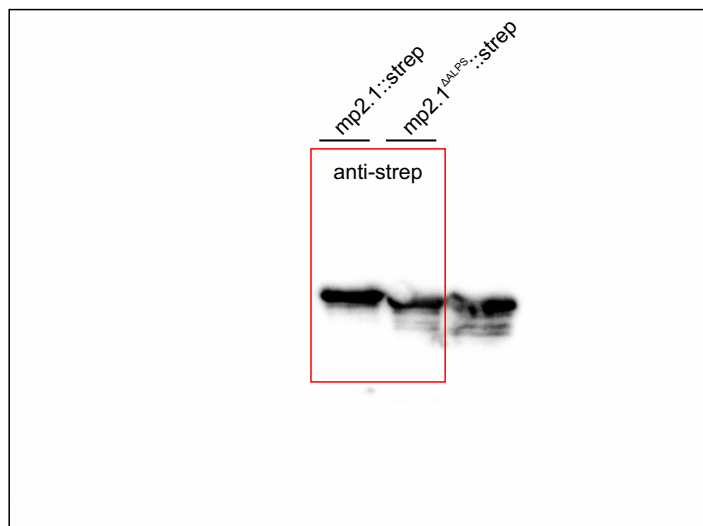

**Fig5 E**

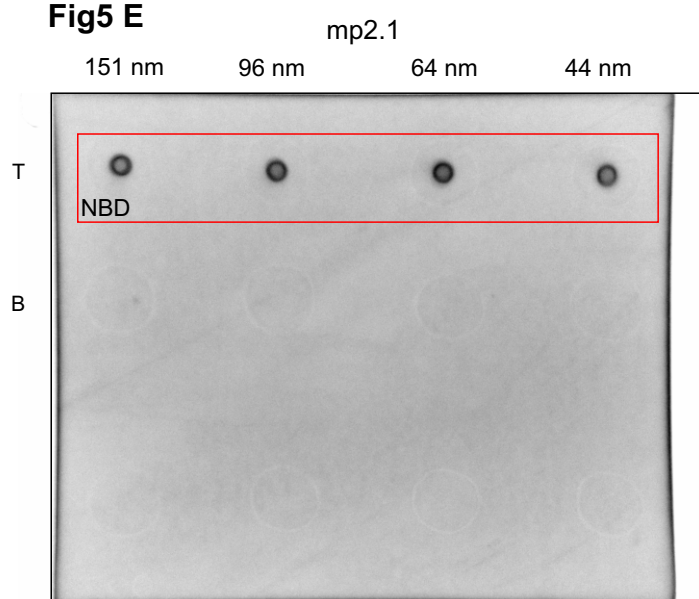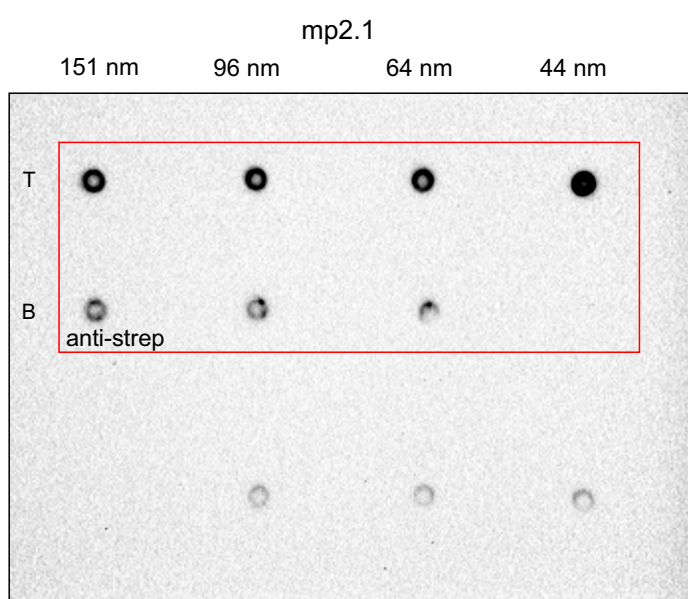

**Fig5 F**

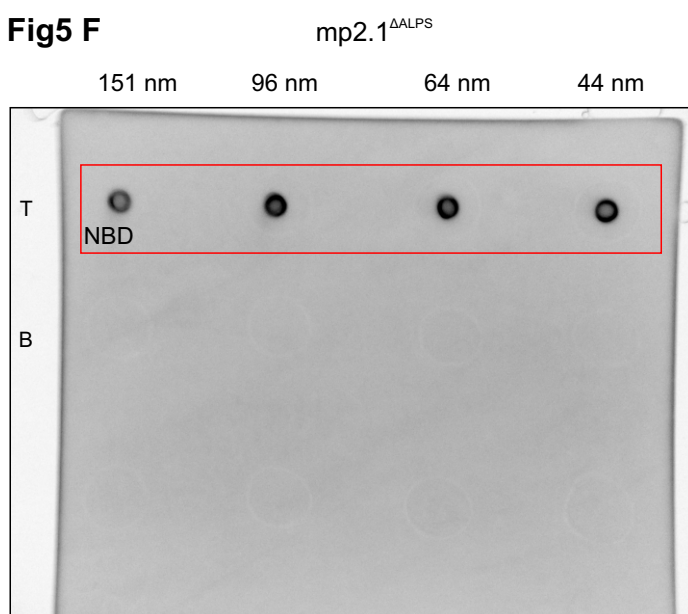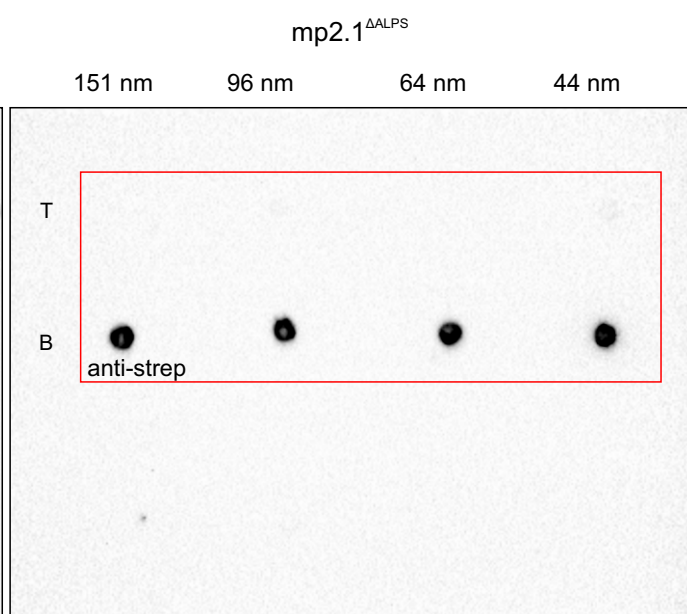

Supplement: SourceData F5 — is the source file for Fig. 5. [file jcb_202509110_sourcedataf5.pdf]

FigS3 B

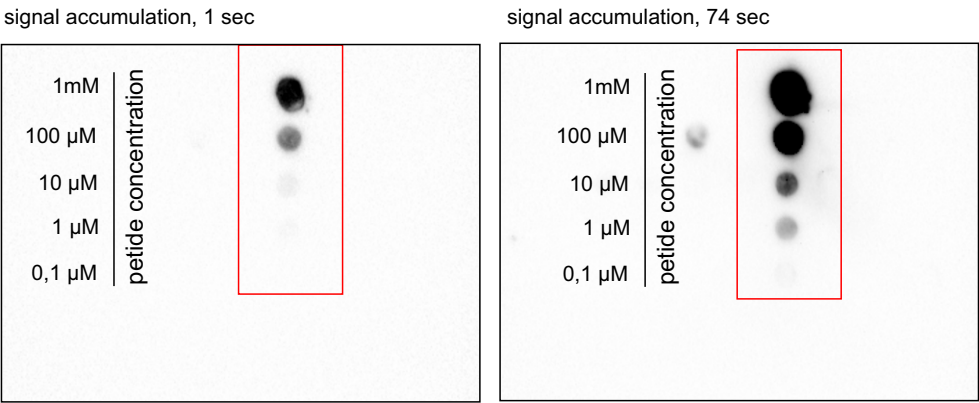

FigS3 C

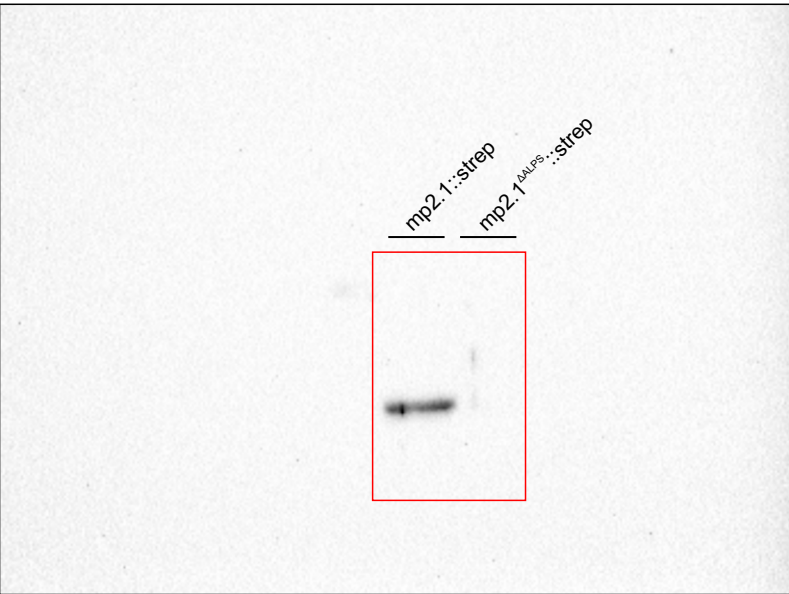

FigS3 D

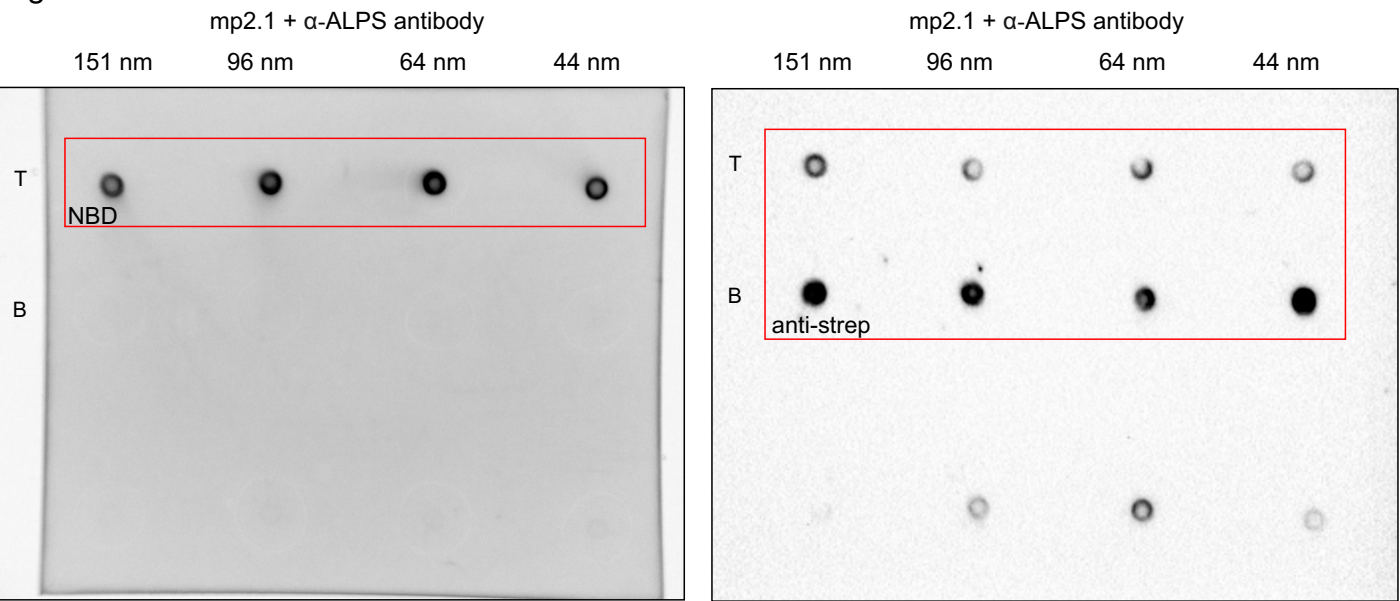

Supplement: SourceData FS3 — is the source file for Fig. S3. [file jcb_202509110_sourcedatafs3.pdf]
