# Supplementary material for: The missing link: Piccolino is essential for tethering synaptic vesicles to rod photoreceptor ribbons
Source: J Cell Biol. 2026 Jul 23;225(9):e202509110. doi: 10.1083/jcb.202509110 (PMC13394108; doi:10.1083/jcb.202509110)
Supplement: Review History [file jcb_202509110_review_history.pdf]

JCB

# The missing link: Piccolino is essential for tethering synaptic vesicles to rod photoreceptor ribbons

Kaspar Gierke, Michalina Gadomska, Julia Breuer, Julius Bahr, Tanja Mülller, Hanna Ehnis, Sina Zobel, Nancy Mejia Villagran, Sonja Kirsch, Alexandra Skrzypek, Renato Frischknecht, Anna Fejtova, Rainer Böckmann, Carolin Wichmann, Hanna Regus-Leidig, and Johann Helmut Brandstätter

*Corresponding Author(s): Kaspar Gierke, Friedrich-Alexander-Universität Erlangen-Nürnberg and Johann Helmut Brandstätter, Friedrich-Alexander-Universität Erlangen-Nürnberg*



## Review Timeline:

| | |
|---|---|
| Submission Date: | 2025-09-12 |
| Editorial Decision: | 2025-10-16 |
| Revision Received: | 2026-05-18 |
| Editorial Decision: | 2026-06-04 |
| Revision Received: | 2026-06-17 |

*Monitoring Editor: Louis Reichardt*

*Scientific Editor: Gabriele Stephan*


## Transaction Report:

(Note: With the exception of the correction of typographical or spelling errors that could be a source of ambiguity, letters and reports are not edited. The original formatting of letters and referee reports may not be reflected in this compilation.)

**DOI: https://doi.org/10.1083/jcb.202509110**

Dr. Kaspar Gierke
Friedrich-Alexander-Universität Erlangen-Nürnberg
Biology, Animal Physiology/Neurobiology
Staudtstr. 5
Erlangen, 91058 Germany
004991318528329
kaspar.gierke@fau.de

October 16, 2025

Re: JCB manuscript 202509110

Dear Dr. Gierke,
I am attaching to this letter the evaluations provided by three reviewers of the manuscript that you submitted recently to this journal. Each of the three reviewers is impressed by the overall high quality of your data. However, there are several major concerns:
1. You have not addressed the possibility that a large fragment of piccolino is expressed in your mutant, potentially acting as a dominant negative. 2. The reviewers do not believe you have shown that piccolino acts itself as a tether binding synaptic vesicles (see reviewer 2 for an alternative interpretation). Both the second and third reviewers favor alternative interpretations. Are the visualized "tethers" acting as tethers or acting to maintain some distance between ribbons and synaptic vesicles? 3. The reviewers have grave reservations about the strength of your conclusions regarding the ALPS domains interactions and the importance of its lipid interactions for localization of synaptic vesicles. To provide a slightly different summary, I consider the first and second reviewers' points 1 and 2 as each crucial. The third reviewer's point 2 seems essential; this reviewer's #3 is similar to one of the second reviewer's points; the reviewer's concerns in #4 about the inconsistency between images and quantification suggests that images selected were not representative. The reviewer expresses in #5 concern about your ability to identify tethers of reduced density. To substantiate your proposed role for the ALPS domain clearly requires substantiation through new experiments and you need to address the apparent inconsistencies between your paper in 2014 vs. the current submission. It seems essential to also investigate the localization of actin in your mutant.

To summarize, the reviewers and I share a strong appreciation for the overall high quality of your data. It seems essential though to provide more convincing evidence for or against your hypothesis that piccolino acts as a tether for synaptic vesicles and to address the possible existence and function of the fragment of piccolino that may be expressed in your mutant and could conceivably explain some of the inconsistencies between this manuscript and your prior publication (since it used RNA interference that seems likely to have reduced expression of all domains of piccolino.

This journal is willing to consider a revised manuscript, but I would caution you against premature resubmission. It will clearly be returned to each of the three reviewers. Continued reservations on their part or parts would result in a final rejection.

Sincerely yours,
Louis F. Reichardt
For the JCB

Gabriele Stephan
Scientific Editor
Journal of Cell Biology

--------------------------------------------------------------------------

Reviewer #1:

Gierke et al use a series of elegant super-resolution imaging, EM, modeling, and biochemistry experiments to provide evidence that the Piccolo splice variant Piccolino is the component of synaptic ribbon synapses responsible for tethering synaptic vesicles to the ribbon. While Piccolino was previously shown to be a component of synaptic ribbons, its specific role has been unclear, and the identity of the SV tether to ribbons has been unknown. Thus, this study provides novel and detailed information about the mechanism of SV tethering at ribbon synapses. Moreover, the quality of imaging and EM data is high, the experiments appear rigorous, and the authors' conclusion is generally convincing. There are nevertheless several issues that need clarification prior to publication of this study, as discussed below.

1. Based on the description of the conditional KO and the diagram in Fig. 1B, it appears as though a large fragment of Piccolino (corresponding to exons 1-4a) would still be expressed in the rod photoreceptor cells, as only the C-terminal region corresponding to exons 4a and 5 is disrupted. If this is the case, it is possible that the truncated Piccolino acts as a dominant-negative and disrupts SV tethering indirectly, e.g. by disrupting the binding of other tethering molecules to SVs. The authors should discuss this possibility, and show whether a truncated fragment of Piccolino is still expressed with the N-terminal antibody used in Fig. 5 (or if expression of the protein is completely abolished).
2. In Figure 2J-L, it appears that some SVs (~12%) are still tethered to the ribbon in Pclo cKO photoreceptor cells. It is not clear how tethering is measured here, as no detailed description is provided. Is tethering in Pclo cKO cells measured solely based on distance between SV and ribbon, or on the presence of a specific morphological structure? If the latter, can the authors comment on what could form these structures in the absence of Piccolino?
3. In Supplemental Figure 2, the shape of the synaptic ribbon does not appear to be altered in Pclo cKO rod photoreceptors when stained with other AZ markers, in contrast to Fig. 1E-G where it is less concave. Can the authors comment on this discrepancy? Also, it would be helpful to have some quantification for this figure (S2) to know what is being measured.
Minor issue: In Figure 2A'-A', the color scheme is a bit confusing. It would be better to keep the colors constant for the SV pools and to indicate what is being measured with dashed red lines encircling the relevant SV pools for A" and A'.

Reviewer #2:

This paper uses sophisticated and elegant methods to investigate the role of Piccolino, a shorter splice variant of the active zone protein Piccolo, at rod photoreceptor synapses. The paper builds on previous studies by the authors demonstrating that the deletion of Piccolo causes a dramatic change in photoreceptor ribbons containing RIBEYE by altering the morphology of the ribbons. The four key findings of the paper are a) the density of SVs on the ribbons is modestly reduced (~20%), b) SVs are closer to the ribbons (average WT 25 nm vs. average KO 14 nm distance), c) 'tethers' visible by EM reconstruction are decreased from 60% to 11% of SVs even though the vesicles are still attached to the ribbons, and d) a lipid-binding motif in the N-termins of Piccolino might bind to SVs. I believe this paper represents a beautiful and important contribution that should be published in the JCB because the data are compelling, but I do not agree with the conclusions of the paper and suggest that it be rewritten.
I have no technical concerns and I agree with the conclusion that Piccolo is likely an essential component of the 'tethers' that are visible by EM. However, I am not convinced by two concepts proposed by the authors.
1) The notion that Piccolino tethers attach SVs to ribbons. Maybe I misunderstood the authors but my interpretation of the data would be that Piccolino is a major component of the physical tethers seen by EM but that these 'tethers' do not actually tether the SVs to the ribbons since the SVs are still attached when the tethers are gone. My interpretation is that most likely the EM-visualized tethers function to keep vesicles at a distance for faster movement down the ribbon. Possibly renaming these tethers as filaments would be better since in the literature SV tethers are usually thought to attach SVs to active zones, which is clearly not the case here.
2) I am not convinced that the N-terminal lipid-binding motif mediates SV recruitment to Piccolino tethers and feel the paper vastly overstates the data here. All that is shown is that Piccolino has an N-terminal lipid-binding motif, no more. It seems to me to be much more likely that Piccolino binds to a vesicle protein. I suggest that this whole part of the manuscript be toned down and deemphasized - it is the gorgeous ultrastructural work that makes this paper significant.
In sum, this paper reports beautiful data that should be published. It seems to me that the very elegant localization of the N- and C-terminus of Piccolo performed here might give a clue to the interesting question of why the Piccolo deletion causes as change in the ribbon morphology: The Piccolo C-terminus might shape the ribbon, which is a very interesting notion that might be discussed.
I hope my comments have been helpful.
Tom Sudhof

Reviewer #3:

The authors used electron microscopy, super-resolution imaging, MD simulation and liposome binding assay to study the function of piccolino in the rod ribbon synapse in mice. Their STED imaging clearly shows that piccolino adopts a fixed orientation at rod ribbon synapse. The EM images of ribbon synapses from piccolino conditional KO mice suggested that piccolino is a potential tethering protein between ribbons and synaptic vesicles (SVs). However, the presented images and the quantification method are not sufficient to support their final conclusion. Some of the data reported in the current study seems to be inconsistent with their previous publication. The morphological change of synaptic ribbons in piccolino mutant mice or after piccolino knockdown has been reported, which also dampens the novelty of this study.

A critical experiment that may allow the authors to obtain more direct evidence to support their conclusion is to re-express a Pclo mutant lacking the short ALPS motif identified to bind to SVs in the Pclo KO mice.

Major issues

1. It is interesting to observe that the rod photoreceptor-specific knockout of Pclo changes the synaptic ribbon shape and size. How may the authors explain that the change of a SV tethering protein also alters the entire SR shape and size? In particular, why would the AZ shape also change?

2. The Pclo cKO was only validated by Pclo 44a immunostaining. Since the authors have several antibodies recognizing different regions of Pclo [1], they should check whether any truncated protein is produced in cKO animals by western-blot and immunostaining. The ALPS motif is located in exon3. It's possible that the truncated protein, if any left, still contains ALPS.

3. It is interesting to note that in Pclo KO photoreceptors, SVs become to be closely attached to SR (Fig. 2G). Does this mean that SVs become directly "docked" onto SRs? If this is the case, what is the role of the tether for SVs if SVs can directly coat on the surface of SRs.

4. From the quantification in Figure 2H, the density of SVs on ribbon surface slightly decreases. But the figure 2B and 2C shows dramatic difference between WT and Pclo cKO retina. A more representative image should be used in Figure 2C. Although the authors claim that the cytoplasmic RP is not affected in cKO group, a decrease of cytoplasmic SV number in Figure 2C is obvious.

5. Figure 2K and 2J is critical for the key conclusion of the manuscript. The authors should be more rigorous when presenting images and performing quantifications. This reviewer is not sure whether the current EM resolution may allow the authors to clearly identify SV tethers shorter than 5 nm. For example, it seems that the ribbons become less electron-condense in Pclo cKO group. How the authors draw the boundary of a ribbon might completely change the quantification result. The dotted line in Figure 2K partially overlaps with the potential tethers, making it hard to see the detail of the ribbon surface. Authors should also present the raw images without dotted lines. Also, more raw images showing different forms of SR structures like in Figure 2K in the supplementary figure will be helpful.

Additionally, why are these filamentous structures (labeled by yellow arrowheads below, the figure is Fig. 3K of the manuscript) not considered as tethers? It's not clear what the criteria are when they quantify the tethers.

6. Figure 1E shows that the ribbon morphology is heterogeneous in Pclo cKO group. Some maintain the horseshoe-like structure and some are fragmented or spherical. The authors should confirm the tethers are lost in all types of ribbons. For example, first classify the ribbons into rod/club/spherical/floating etc. and then perform the quantification.

7. The data presented in the manuscript are not consistent with their previous publication [2]. The EM images below are the rod synaptic terminals infected by AAV-pclo shRNA in the previous paper. The morphological changes of ribbons are quite consistent with the current manuscript. However, numerous SV tethers can be found around the ribbon (labeled by yellow arrowheads). The distance between SVs and ribbons appears not affected. Authors should explain the discrepancies of the two papers.

8. Related to the above issue and given past and recent literatures suggesting that actin filaments may serve as tethers for SVs, the authors should investigate possible roles of actin filaments in tethering SVs to SR.

9. The liposome floatation assay is the only experiment for the validation of protein-lipid interaction. More biochemical or cell-based assays are needed.
(9a) In Fig. 5B, it is essential to show the full SDS-PAGE of purified Pclo fragment with TCE staining, to make sure that the qualities of the proteins are sufficiently high.
(9b) It is curious that for mp2.1 and particularly for small liposomes, all proteins are bound to liposomes. This is very striking and unusual. One expects that the ALPS motif-mediated lipid binding is generally weak.
(9c) Does Pclo ALPS interact with a specific type of lipid? Lipid strips can be used to demonstrate the specificity of the protein-lipid interactions.
(9d) Does Pclo ALPS recruit small vesicles in non-neuronal cells? Authors can design a chimeric Pclo construct, for example, a protein targeting Pclo ALPS to mitochondria and check if the protein also tethers vesicles to mitochondria by EM.

Minor issues:
1. The manuscript only investigates the role of piccolino in rod photoreceptors. If piccolino is the general tethering factor for all the ribbon synapses, other cKO models (cone, bipolar and IHC) should be checked as well.

2. SV clustering in CNS presynapse has been extensively studied. In addition to piccolo, previous literature demonstrated that F-actin and synapsin were the potential tethering factors for SV clustering. Immunogold labeling can be used to check if these proteins are co-localize with synaptic ribbons.

3. In other CNS presynapse, long Pclo variant not only contains the N-terminal ALPS but also the C-terminal C2 domains. Is Pclo a possible candidate for tethering two SVs simultaneously in other CNS synapse?

4. The authors prepared SV-like liposomes with different diameters without any validation. The sizes of liposomes need to be confirmed by EM.

[1] Regus-Leidig, H., Ott, C., Löhner, M., Atorf, J., Fuchs, M., Sedmak, T., ... & Brandstätter, J. H. (2013). Identification and immunocytochemical characterization of Piccolino, a novel Piccolo splice variant selectively expressed at sensory ribbon synapses of the eye and ear. PloS one, 8(8), e70373.
[2] Regus-Leidig, H., Fuchs, M., Löhner, M., Leist, S. R., Leal-Ortiz, S., Chiodo, V. A., ... & Brandstätter, J. H. (2014). In vivo knockdown of Piccolino disrupts presynaptic ribbon morphology in mouse photoreceptor synapses. Frontiers in cellular neuroscience, 8, 259.

Dear Editor-in-Chief Jodi Nunnari, Dear Gabriele Stephan, Dear Louis Reichardt,

Please find enclosed our revised manuscript (tracking number 202509110) for your consideration. The three Reviewers' valuable and constructive comments are gratefully acknowledged and we have made a major effort to implement all their suggestions. By performing new experiments and adding new data, the revised manuscript includes improved figures (Figs. 1, 2, 5 and supplementary Figs. 2 and 3), as well as extensive changes and additions to the text. We trust that these changes have substantially improved the manuscript and hope that you and the Reviewers will now consider it suitable for publication in *The Journal of Cell Biology*. Below, please find our point-by-point responses to the Reviewers' concerns and suggestions.

Reviewer #1:

Gierke et al use a series of elegant super-resolution imaging, EM, modeling, and biochemistry experiments to provide evidence that the Piccolo splice variant Piccolino is the component of synaptic ribbon synapses responsible for tethering synaptic vesicles to the ribbon. While Piccolino was previously shown to be a component of synaptic ribbons, its specific role has been unclear, and the identity of the SV tether to ribbons has been unknown. Thus, this study provides novel and detailed information about the mechanism of SV tethering at ribbon synapses. Moreover, the quality of imaging and EM data is high, the experiments appear rigorous, and the authors' conclusion is generally convincing. There are nevertheless several issues that need clarification prior to publication of this study, as discussed below.

We thank the Reviewer for the positive feedback and the constructive criticism.

1. Based on the description of the conditional KO and the diagram in Fig. 1B, it appears as though a large fragment of Piccolino (corresponding to exons 1-4a) would still be expressed in the rod photoreceptor cells, as only the C-terminal region corresponding to exons 4a and 5 is disrupted. If this is the case, it is possible that the truncated Piccolino

acts as a dominant-negative and disrupts SV tethering indirectly, e.g. by disrupting the binding of other tethering molecules to SVs. The authors should discuss this possibility, and show whether a truncated fragment of Piccolino is still expressed with the N-terminal antibody used in Fig. 5 (or if expression of the protein is completely abolished).

A truncated Piccolino that acts as a dominant-negative is possible from the cloning strategy. To address this issue, we performed two experiments:

1. As suggested by the Reviewer, we performed stainings with the N-terminal Pclo4 antibody. No staining of a truncated Piccolino variant could be detected in rod photoreceptors. In the revised Figs. 1 C,D, we replaced the previous Pclo44a antibody staining with the N-terminal Pclo4 antibody staining.

2. In a second experiment, we performed a Western Blot using the N-terminal Pclo4 antibody. This also did not show a truncated Pclo fragment (new Fig. 1 E). Notably, only a mild reduction in Piccolino was observed. This is due to its presence in cone photoreceptors and bipolar cells. Together, these results argue against a dominant-negative effect of a truncated Piccolino fragment. The findings have been added to Materials and Methods (lines 527-542) and the Results (lines 680-687).

2. In Figure 2J-L, it appears that some SVs (~12%) are still tethered to the ribbon in Pclo cKO photoreceptor cells. It is not clear how tethering is measured here, as no detailed description is provided. Is tethering in Pclo cKO cells measured solely based on distance between SV and ribbon, or on the presence of a specific morphological structure?

We apologize for forgetting to explain how tethering was measured. A detailed explanation has been added to the Materials and Methods section (lines 632-640).

Briefly, in the conventional TEM images (60 kV) (Figs. 2 B-E), we solely used the distance between SVs and ribbon as an indirect measure of tethered SVs, because filaments could not be resolved. In the higher power EM tomograms (200 kV), with a resolution in the nanometer range, (Figs. 2 J-M’), we only counted SVs as tethered, when they were in contact with the SR via a filament (indicated by the black arrowheads in Fig. 2 L). SVs in direct contact with the ribbon were not counted as tethered because they lacked a filament.

2.1 If the latter, can the authors comment on what could form these structures in the absence of Piccolino?

Following possible explanations for the presence of some tethered SVs in Pclo cKO rod photoreceptor ribbon synapses have been added to the discussion in the revised manuscript (lines 1027-1038):

1. The late onset of the Cre-Driver at around postnatal day 6. This is after the onset of Piccolino expression, which can be detected as early as postnatal day 2 (Regus-Leidig et al., 2009). It is thus possible that a few copies of Piccolino are present in the synapse, forming tethers in $Pclo^{cKO}$ rod photoreceptor ribbon synapses.

2. In a few cases, it was not possible to delineate the SR border with 100 % accuracy in the $Pclo^{cKO}$ rod photoreceptors. This is because the higher accelerating voltage of the high-power TEM (200 kV) increases beam penetration, which reduces image contrast at the ribbon border. In these cases, it is difficult to discriminate the ribbon border from putative tethers. To be as unbiased as possible, we included them in the "tethered" fraction. However, given the significant decrease of tethered SVs in the $Pclo^{cKO}$ rod photoreceptor ribbon synapses, our data suggest that Piccolino constitutes the majority of filaments observed at WT rod photoreceptor ribbon synapses.

3. In Supplemental Figure 2, the shape of the synaptic ribbon does not appear to be altered in Pclo cKO rod photoreceptors when stained with other AZ markers, in contrast to Fig. 1E-G where it is less concave. Can the authors comment on this discrepancy?

To show the presence of the AZ proteins, we selected the best images possible. We acknowledge that this may, in some cases, not properly reflect the phenotype shown in Fig 1. Accordingly, we replaced the current high-power views for ubMunc13-2 and $Ca_V1.4$ with examples better reflecting the overall cKO phenotype.

Regarding the concavity, we think that the apparent decrease in curvature is not a separate phenotype, but due to the fact that the $Pclo^{cKO}$ rod photoreceptor ribbons are shorter.

3.1 Also, it would be helpful to have some quantification for this figure (S2) to know what is being measured.

Nothing was quantified or measured in Supplemental Figure 2 because we only wanted to show qualitatively that all other SR and AZ proteins retain their localization absent Piccolino. This showed us that the observed phenotypes are specific to Piccolino.

3.2 Minor issue: In Figure 2A'-A', the color scheme is a bit confusing. It would be better to keep the colors constant for the SV pools and to indicate what is being measured with dashed red lines encircling the relevant SV pools for A" and A'.

Thank you for pointing this out. We now keep the colors constant throughout A-A''' and mark what is being measured with red dashed lines.

Reviewer #2:

This paper uses sophisticated and elegant methods to investigate the role of Piccolino, a shorter splice variant of the active zone protein Piccolo, at rod photoreceptor synapses. The paper builds on previous studies by the authors demonstrating that the deletion of Piccolo causes a dramatic change in photoreceptor ribbons containing RIBEYE by altering the morphology of the ribbons. The four key findings of the paper are a) the density of SVs on the ribbons is modestly reduced (~20%), b) SVs are closer to the ribbons (average WT 25 nm vs. average KO 14 nm distance), c) 'tethers' visible by EM reconstruction are decreased from 60% to 11% of SVs even though the vesicles are still attached to the ribbons, and d) a lipid-binding motif in the N-terminus of Piccolino might bind to SVs. I believe this paper represents a beautiful and important contribution that should be published in the JCB because the data are compelling, but I do not agree with the conclusions of the paper and suggest that it be rewritten.

We thank the Reviewer for the positive feedback and the constructive criticism.

I have no technical concerns and I agree with the conclusion that Piccolo is likely an essential component of the 'tethers' that are visible by EM. However, I am not convinced by two concepts proposed by the authors.

1. The notion that Piccolino tethers attach SVs to ribbons. Maybe I misunderstood the authors but my interpretation of the data would be that Piccolino is a major component of the physical tethers seen by EM but that these 'tethers' do not actually tether the SVs to the ribbons since the SVs are still attached when the tethers are gone.

It is true that SVs are still found on the surface of SRs in the absence of Piccolino. This could create the impression that a tether is not necessary to attach SVs to the SR. However, we have now extended our arguments in the discussion of the revised manuscript that speak against this notion. Firstly, the quantification of SR-tethered SVs clearly shows that SV density decreases in the absence of Piccolino. Secondly, ribbon synapse terminals are filled with thousands of freely diffusible SVs (Holt et al., 2004), making it highly probable that they will come into contact with SRs by chance alone. Additionally, we included the argument that the main function of a tether is not the attachment of SVs to SRs *per se*, but to capture, position and transport them to the AZ. We question whether these SVs, which appear in direct contact with SRs are physiologically relevant and that their targeted transport to the AZ is possible in the absence of a tether (lines 1017-1025).

1.1. My interpretation is that most likely the EM-visualized tethers function to keep vesicles at a distance for faster movement down the ribbon. Possibly renaming these tethers as filaments would be better since in the literature SV tethers are usually thought to attach SVs to active zones, which is clearly not the case here.

The Reviewer is absolutely right; SV tethers attach SVs to the active zone. In the field of sensory ribbon synapses (eye and ear), the SR is considered an extension of the active zone. Therefore, in analogy to conventional chemical synapses, the term SV tether is used to refer to the filaments between SVs and SR.

To clarify the similarities and differences between conventional and sensory ribbon synapses with respect to the active zone and SV-tethering, we substantially reworked the introduction. Specifically, we added a new section that focuses on SV tethering from conventional synapses. We now clearly distinguish the molecules and potential mechanisms involved in conventional and ribbon synapses. Additionally, we explicitly state what the role of the tethering process might be in ribbon synapses. We share the Reviewers' view that the ribbon tethers function to keep vesicles at a distance for faster movement down the ribbon.

We hope that these changes without replacing the term tether, are an acceptable solution for the Reviewer.

2. I am not convinced that the N-terminal lipid-binding motif mediates SV recruitment to Piccolino tethers and feel the paper vastly overstates the data here. All that is shown is that Piccolino has an N-terminal lipid-binding motif, no more. It seems to me to be much more likely that Piccolino binds to a vesicle protein. I suggest that this whole part of the manuscript be toned down and deemphasized.

It was also our initial hypothesis that Piccolino interacts with a vesicle protein, such as Rab3A. However, all our attempts to uncover a protein-protein interaction failed, forcing us to look for alternative mechanisms. While our data may not provide definitive proof of ALPS-mediated tethering *in cellulo*, they do provide a strong working hypothesis consistent with all our observations. Accordingly, we toned down the significance of this finding by revising the wording in the results (lines 888-892) and the discussion (lines 963; lines 1002-1005). At the same time, we strengthened our hypothesis by including a cell-based assay showing that the ALPS-containing mp2.1 fragment is able to recruit endogenous SVs in cultured cortical neurons. The findings have been added to supplementary Fig. 3 E-F, Materials and Methods (lines 187-191; lines 272-291; lines 414-426; lines 435-448), and the Results (lines 874-887).

- it is the gorgeous ultrastructural work that makes this paper significant.

We thank the Reviewer for appreciating our ultrastructural work.

In sum, this paper reports beautiful data that should be published. It seems to me that the very elegant localization of the N- and C-terminus of Piccolo performed here might give a clue to the interesting question of why the Piccolo deletion causes as change in the ribbon morphology: The Piccolo C-terminus might shape the ribbon, which is a very interesting notion that might be discussed.

Once again, we thank the Reviewer for the kind words.  The Reviewer's notion that the deletion of Piccolino causes a change in ribbon morphology has already been explored in a previous study (Müller et al., 2019). Here, we demonstrated that the C-terminus of Piccolino interacts with RIBEYE via multiple PxDLS-like motifs, thereby stabilizing the ribbon superstructure. We have added a sentence in the discussion to make this connection more explicit (lines 951-957).

I hope my comments have been helpful.

Thank you, they have been very helpful.

Reviewer #3

The authors used electron microscopy, super-resolution imaging, MD simulation and liposome binding assay to study the function of piccolino in the rod ribbon synapse in mice. Their STED imaging clearly shows that piccolino adopts a fixed orientation at rod ribbon synapse. The EM images of ribbon synapses from piccolino conditional KO mice suggested that piccolino is a potential tethering protein between ribbons and synaptic vesicles (SVs). However, the presented images and the quantification method are not sufficient to support their final conclusion. Some of the data reported in the current study seems to be inconsistent with their previous publication. The morphological change of synaptic ribbons in piccolino mutant mice or after piccolino knockdown has been reported, which also dampens the novelty of this study.

We thank the Reviewer for the feedback and the constructive criticism.

A critical experiment that may allow the authors to obtain more direct evidence to support their conclusion is to re-express a Pclo mutant lacking the short ALPS motif identified to bind to SVs in the Pclo KO mice.

We agree that this would be the gold standard experiment to unequivocally prove our hypothesis. However, the necessary experiments are beyond the scope of this study, both technically – involving subretinal injection of lentiviral particles harboring a ~ 9 kb construct – and in terms of the currently available regulatory framework for animal experiments in our lab.

Major issues

1. It is interesting to observe that the rod photoreceptor-specific knockout of Pclo changes the synaptic ribbon shape and size. How may the authors explain that the change of a SV tethering protein also alters the entire SR shape and size?

We demonstrated in a previous study that the C-terminus of Piccolino interacts with RIBEYE via multiple PxDLS-like motifs, thereby stabilizing the ribbon superstructure (Müller et al., 2019). In this paper, we proposed that the N-terminus of Piccolino may extend into the cytosol to capture SVs (this study). In conclusion, based on our previous and this study, we propose that the C- and N-terminal domains of Piccolino have distinct functions: shaping the ribbon structure and tethering SVs, respectively.

1.1. In particular, why would the AZ shape also change?

It has been demonstrated that the SR and the AZ compartment are co-regulated: the removal of the SR affects AZ size (Maxeiner et al., 2016), and vice versa, the deletion of AZ proteins affects SR size (tom Dieck et al., 2012, Hagiwara et al., 2018). To our knowledge, the mechanism of this co-regulation is not yet understood.

2. The Pclo cKO was only validated by Pclo 44a immunostaining. Since the authors have several antibodies recognizing different regions of Pclo [1], they should check whether any truncated protein is produced in cKO animals by western-blot and immunostaining. The ALPS motif is located in exon3. It's possible that the truncated protein, if any left, still contains ALPS.

Thank you for pointing this out. We copy-paste the response to comment #1 of Reviewer #1, who has raised the same concern:

A truncated Piccolino acting dominant-negative is possible from the cloning strategy, and we performed two experiments to address this issue.

1. As suggested by the Reviewer, we performed immunostainings with the N-terminal Pclo4 antibody. No residual staining pointing towards a truncated Piccolino variant could be detected in rod photoreceptors. Because, this antibody recognizes all Pclo/Piccolino variants detected by Pclo44a we have replaced the Pclo44a antibody staining in Fig. 1°C-D with the N-terminal Pclo4 antibody staining.

2. As a more sensitive method, we performed a Western Blot using the Pclo4 antibody (new Panel Fig. 1 E), which previously detected a fragment in the Pclo$^{gt/gt}$ rat model (Müller et al., 2019). We could not detect a residual, truncated Pclo fragment. Notably, only a mild reduction in Piccolino was observed due to the presence of Piccolino in cone photoreceptor and bipolar cell terminals. Together, these results argue against a dominant-negative effect of a truncated Piccolino fragment. The findings have been added to Materials and Methods (lines 528-542) and the Results (lines 680-687)

3. It is interesting to note that in Pclo KO photoreceptors, SVs become to be closely attached to SR (Fig. 2G). Does this mean that SVs become directly "docked" onto SRs? If this is the case, what is the role of the tether for SVs if SVs can directly coat on the surface of SRs.

It is true that SVs are found on the surface of SRs in the absence of Piccolino. This could create the impression that SVs become directly “docked” onto SRs. However, we have now included arguments in the discussion of the revised manuscript that speak against

this notion. Firstly, the quantification of SR-tethered SVs clearly shows that SV density decreases in the absence of Piccolino. Secondly, terminals of ribbon synapses are filled with thousands of freely diffusible SVs (Holt et al., 2004), making it highly probable that they will come into contact with SRs by chance alone. It is questionable whether these directly bound SVs are physiologically relevant and whether their targeted transport to the active zone is possible in the absence of a tether. (lines 1017-1025)

4. From the quantification in Figure 2H, the density of SVs on ribbon surface slightly decreases. But the figure 2B and 2C shows dramatic difference between WT and Pclo cKO retina. A more representative image should be used in Figure 2C. Although the authors claim that the cytoplasmic RP is not affected in cKO group, a decrease of cytoplasmic SV number in Figure 2C is obvious.

We have replaced the EM micrographs in Figure 2 with images that more accurately represent the quantitative data relating to both the density of SVs close to the ribbon and the density of SVs in the cytoplasmic pool.

5. Figure 2K and 2J is critical for the key conclusion of the manuscript. The authors should be more rigorous when presenting images and performing quantifications. This reviewer is not sure whether the current EM resolution may allow the authors to clearly identify SV tethers shorter than 5 nm.

There seems to be a misunderstanding. The Reviewer is right that our approach would miss SV tethers shorter than 5 nm. However, we were not looking for such short tethers, but for tethers that span a distance of 25-35 nm between the SVs and the SR. This is the distance that the N-terminus of Piccolino extends into the cytoplasm, and which can be resolved using electron tomography that is capable to visualize structures with a resolution of ~1 nm.

5.1 For example, it seems that the ribbons become less electron-condense in Pclo cKO group. How the authors draw the boundary of a ribbon might completely change the quantification result.

It is correct that delineating the border in tomograms isn't straightforward, because the electron beam of a 200 kV microscope penetrates the sample more readily, contributing to the reduction of the contrast at the ribbon boundary. However, by reconstructing the ribbon frame-by-frame, we can reliably distinguish between the ribbon material and the tethers, which are only visible in ~ 3-5 sections. To ensure that the reduced electron density is not an artifact of the HPF procedure, we have also checked ultrathin sections of the HPF tissue with our conventional 60 kV TEM. In these images, the boundary of the ribbon is easily visible due to the lower acceleration voltage, and the SV phenotype (i.e., decreased SV density and SV distance to the SR) is consistent with our other data. We have included the images of HPF-treated tissue in the revised Fig. 2 (Figs. 2 J,K).

5.2 The dotted line in Figure 2K partially overlaps with the potential tethers, making it hard to see the detail of the ribbon surface. Authors should also present the raw images without dotted lines

We have removed the dotted lines high-power views in Fig. 2 L and 2 M (previous Fig. 2K,L) for improved visibility of the ribbon border.

5.3 Also, more raw images showing different forms of SR structures like in Figure 2K in the supplementary figure will be helpful.

We decided not to show more raw images of different SR forms, because only spherical aggregates were analyzed for the SV density and distance analysis. The reason is that we excluded the first 100 nm of ribbon material, i.e., they part of the ribbon that is located in the arciform density, to avoid confusion between SVs tethered to the SR and the AZ (see lines 627-629 and schematic in Fig 2 A').

5.4 Additionally, why are these filamentous structures (labeled by yellow arrowheads below, the figure is Fig. 3K of the manuscript) not considered as tethers? It's not clear what the criteria are when they quantify the tethers.

We only looked at tethers originating from synaptic ribbons. We have addressed this by extending the materials and methods sections (please also see comment #2, Reviewer #1).

6. Figure 1E shows that the ribbon morphology is heterogeneous in Pclo cKO group. Some maintain the horseshoe-like structure and some are fragmented or spherical. The authors should confirm the tethers are lost in all types of ribbons. For example, first classify the ribbons into rod/club/spherical/floating etc. and then perform the quantification.

We did not categorize the ribbons for the following reasons:

1) The categorization in rod/club/spherical phenotypes comes from the analysis in 2D. In 2D, the appearance of ribbons in a given TEM image depends on the section plane. Thus, they represent different parts of the same ribbon, not separate phenotypes. Since this is not an issue in our 3D tomography analysis, we do not believe that a separate classification would provide additional information.
2) There are virtually no floating or club-shaped ribbons in the $Pclo^{cKO}$ rod photoreceptors. Together, they comprise < 1% of all ribbons (data not shown), which is consistent with the results from a previous study (Regus-Leidig et al., 2014)

7. The data presented in the manuscript are not consistent with their previous publication [2]. The EM images below are the rod synaptic terminals infected by AAV-pclo shRNA in the previous paper. The morphological changes of ribbons are quite consistent with the current manuscript. However, numerous SV tethers can be found around the ribbon (labeled by yellow arrowheads). The distance between SVs and ribbons appears not affected. Authors should explain the discrepancies of the two papers.

A comparison of the results of the ribbon and tethering phenotype between our previous publication (Regus-Leidig et al., 2014) and the present study requires careful consideration of the different experimental approaches employed. In our previous publication, we analyzed a RNAi-mediated knockdown of Piccolino with variable (and unknown) knockdown efficiency. Since Piccolino supports plate-shaped synaptic

ribbons (Müller et al., 2019), a partial knockdown is likely sufficient destabilize the ribbon. It is unclear whether a partial Piccolino knockdown is sufficient to cause a clear SV tethering defect. We have included a separate paragraph in the discussion that explains the apparent differences between this publication and our previous one (lines 919-924).

8. Related to the above issue and given past and recent literatures suggesting that actin filaments may serve as tethers for SVs, the authors should investigate possible roles of actin filaments in tethering SVs to SR.

In conventional chemical synapses, actin filaments tether SVs via interaction with Synapsins, which maintains the reserve pool of SVs. However, this does not seem to be the case in photoreceptor ribbon synapses. Firstly, Synapsins are absent from photoreceptor ribbon synapses of several vertebrates, including mice (Mandell et al., 1990, Geppert et al., 1994, Hirano et al., 2005). Secondly, we visualized F-actin (Phalloidin staining) in rod photoreceptors together with RIBEYE (rebuttal Fig. 1 A). The staining suggests that F-actin is strongly present postsynaptic to photoreceptors (white arrowheads, rebuttal Fig. 1 A). In contrast, it is barely detectable in the presynaptic terminal, particularly near synaptic ribbons as seen in the high-power views (rebuttal Fig. 1 A'). These findings are in line with previous studies showing that SVs in ribbon-type synapses are, unlike at conventional synapses, freely diffusible (Holt et al., 2004) and that tethers emanating from SRs were not labelled by actin antibodies (Usukura and Yamada, 1987).

9. The liposome floatation assay is the only experiment for the validation of protein-lipid interaction. More biochemical or cell-based assays are needed.

Following the suggestion of the Reviewer, we generated a Piccolino mp2.1 fragment fused N-terminally to mCherry as a fluorescent reporter and C-terminally to a mitochondria-targeting sequence (mitoTag) (mCherry::mp2.1::mitoTag), and an equivalent construct carrying the ΔALPS mutation (mCherry::mp2.1$^{\Delta ALPS}$::mitoTag). These constructs were delivered to primary cortical neurons via AAV-mediated infection

for heterologous expression. Neurons were then processed for triple immunofluorescence staining using mCherry signal (construct localization), Grp75 (mitochondrial marker), and vGLUT1 (synaptic vesicle marker). We quantified targeting of our constructs to mitochondria by calculating the Pearson's correlation coefficient between the Grp75 and mCherry channels. We quantified mitochondria–vesicle co-localization by calculating the Pearson's correlation coefficient between the Grp75 and vGGLUT1 channels. To avoid confounding the analysis with synaptic mitochondria, which are naturally in close vicinity to synaptic vesicles, we restricted our analysis to somatic mitochondria. Neurons expressing the ΔALPS construct showed reduced co-localization compared to the wild-type fragment, consistent with the results from our floatation assays and MD simulations. The findings have been added to supplementary Fig. 3 E-F, Materials and Methods (lines 187-191; lines 272-291; lines 414-426; lines 435-448), and the Results (lines 874-887).

9.1 In Fig. 5B, it is essential to show the full SDS-PAGE of purified Pclo fragment with TCE staining, to make sure that the qualities of the proteins are sufficiently high.

We have attached a figure to this letter showing the uncropped SDS gel (rebuttal Fig. 2 A) and Western blot shown in Fig. 5 B (rebuttal Fig. 2 A'). Additionally, we show a TCE-stained SDS gel capturing all steps of the Strep column purification (rebuttal Fig. 2 B). Due to the limited space, we did not add them to Fig. 5. However, we will include uncropped images in the source file required for the final version of the manuscript.

9.2 It is curious that for mp2.1 and particularly for small liposomes, all proteins are bound to liposomes. This is very striking and unusual. One expects that the ALPS motif-mediated lipid binding is generally weak.

A very high degree of binding (> 90 %) is reported in the literature when floatation assays are used (Bigay et al., 2005). In this purified system, the protein is exposed only to the supplied lipid bilayers in ~ 1000-fold excess. Additionally, in this artificial system no competing interactions are present that may occur in cellular environments. In this

setup, even relatively weak interactions can result in efficient co-fractionation. Thus, strong flotation of the protein and a low KD are not mutually exclusive.

9.3 Does Pclo ALPS interact with a specific type of lipid? Lipid strips can be used to demonstrate the specificity of the protein-lipid interactions.

We only touched on this briefly in the manuscript, but ALPS do not interact with a specific lipid. Instead, they interact non-specifically with hydrophobic tails when the packing defects, i.e., the space between phospholipid headgroups become sufficiently large. This can be achieved by (i) decreasing the diameter of a lipid (this splays the outer leaflet) (ii) increasing the amount of lipids with a conical headgroup (this increases the space between headgroups), or (iii) by increasing lipids that act as a wedge between lipids (e.g., cholesterol). This is why our MD simulation show much weaker binding when a pure PC membrane is used (Fig. 4 C). To make this more comprehensible to readers that are not familiar with ALPS motifs, we extended the introduction to ALPS motifs (lines 804-807).

9.4 Does Pclo ALPS recruit small vesicles in non-neuronal cells? Authors can design a chimeric Pclo construct, for example, a protein targeting Pclo ALPS to mitochondria and check if the protein also tethers vesicles to mitochondria by EM.

We refer the Reviewer to our response to point # 9, where we performed the experiment suggested by the reviewer, albeit in cortical neurons.

Minor issues:

1. The manuscript only investigates the role of piccolino in rod photoreceptors. If piccolino is the general tethering factor for all the ribbon synapses, other cKO models (cone, bipolar and IHC) should be checked as well.

It would certainly be interesting to determine whether Piccolino is the general tethering factor for all ribbon synapses. However, this is beyond the scope of this study, which focuses on the rod photoreceptor ribbon synapse.

2. SV clustering in CNS presynapse has been extensively studied. In addition to piccolo, previous literature demonstrated that F-actin and synapsin were the potential tethering factors for SV clustering. Immunogold labeling can be used to check if these proteins are co-localize with synaptic ribbons.

Please see our response to Major Point #8, which also addresses this issue.

3. In other CNS presynapse, long Pclo variant not only contains the N-terminal ALPS but also the C-terminal C2 domains. Is Pclo a possible candidate for tethering two SVs simultaneously in other CNS synapse?

Yes, we believe that this is the case, especially because a second ALPS motif is predicted to be located in the region of the C2 domains (see Suppl. S1 in (Drin et al., 2007)). Separately, the C-terminal region has been shown to be involved in SV tethering via phase separation (Qiu et al., 2024).

4. The authors prepared SV-like liposomes with different diameters without any validation. The sizes of liposomes need to be confirmed by EM.

It is technically challenging to visualize protein-free liposomes in EM because, unlike SVs isolated from brain, liposomes lack free amino groups required for formaldehyde fixation. As an alternative method, we performed dynamic light scattering (DLS) to determine the liposome sizes after extrusion. We have added the findings to the revised Fig. 5 (Fig. 5 C), Material and Methods (lines 495-500) and the Results (857-859).

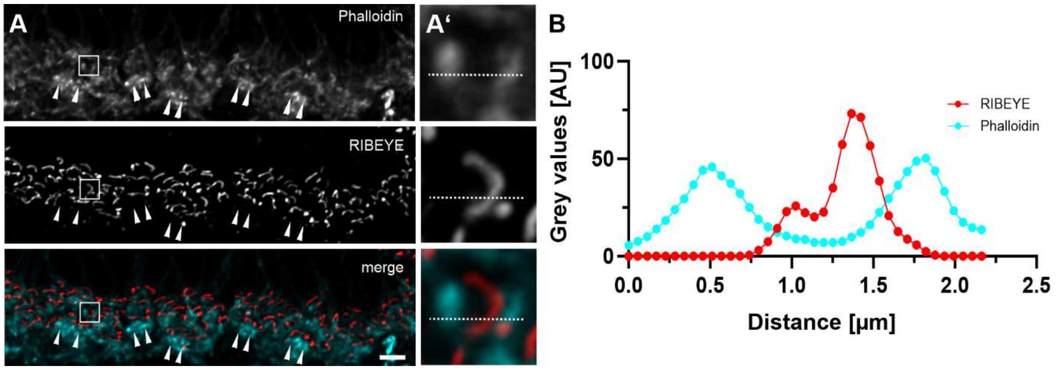


Rebuttal Figure 1. **A,A'**) Confocal images of the outer plexiform layer (OPL) of a wildtype retina labelled with phalloidin (F-actin, cyan), and RIBEYE (red). Arrowheads indicate actin clusters postsynaptic to cone photoreceptors. **B**) Line intensity profile plots along the dashed line in high-power views (A) are shown. Scale bar: 5 µm.

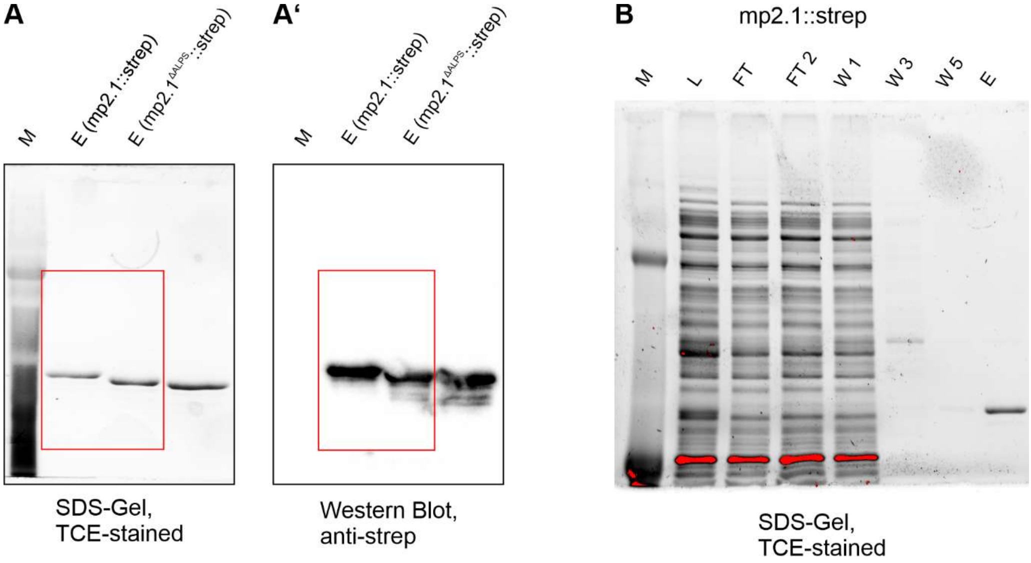


Rebuttal Figure 2. Purification of recombinant mp2.1::strep and mp2.1$^{\Delta ALPS}$::strep from E. coli via strep-tag affinity chromatography. **A,A'**) Uncropped images from manuscript fig. 5: TCE-stained SDS-PAGE of purified mp2.1::strep and mp2.1$^{\Delta ALPS}$::strep after buffer exchange (A) and the corresponding Western blot with anti-strep antibodies (A'). Red rectangle indicates cropping site. **B**) TCE-stained SDS-PAGE of fractions collected during strep-column purification. M, marker; L, lysate; FT, flow-through; W1–W3, wash steps; E, eluate.

References

1. Bigay, J., J.F. Casella, G. Drin, B. Mesmin, B. Antonny. Arfgap1 Responds to Membrane Curvature through the Folding of a Lipid Packing Sensor Motif. *EMBO J* **24**, 2244-2253, doi:10.1038/sj.emboj.7600714 (2005).
2. Drin, G., J.F. Casella, R. Gautier, T. Boehmer, T.U. Schwartz, B. Antonny. A General Amphipathic Alpha-Helical Motif for Sensing Membrane Curvature. *Nat Struct Mol Biol* **14**, 138-146, doi:10.1038/nsmb1194 (2007).
3. Geppert, M., B. Ullrich, D.G. Green, K. Takei, L. Daniels, P. De Camilli, T.C. Sudhof, R.E. Hammer. Synaptic Targeting Domains of Synapsin I Revealed by Transgenic Expression in Photoreceptor Cells. *EMBO J* **13**, 3720-3727, doi:10.1002/j.1460-2075.1994.tb06681.x (1994).
4. Hagiwara, A., Y. Kitahara, C.P. Grabner, C. Vogl, M. Abe, R. Kitta, K. Ohta, K. Nakamura, K. Sakimura, T. Moser, A. Nishi, T. Ohtsuka. Cytomatrix Proteins Cast and Elks Regulate Retinal Photoreceptor Development and Maintenance. *J Cell Biol* **217**, 3993-4006, doi:10.1083/jcb.201704076 (2018).
5. Hirano, A.A., J.H. Brandstatter, N.C. Brecha. Cellular Distribution and Subcellular Localization of Molecular Components of Vesicular Transmitter Release in Horizontal Cells of Rabbit Retina. *J Comp Neurol* **488**, 70-81, doi:10.1002/cne.20577 (2005).
6. Holt, M., A. Cooke, A. Neef, L. Lagnado. High Mobility of Vesicles Supports Continuous Exocytosis at a Ribbon Synapse. *Curr Biol* **14**, 173-183, doi:10.1016/j.cub.2003.12.053 (2004).
7. Mandell, J.W., E. Townes-Anderson, A.J. Czernik, R. Cameron, P. Greengard, P. De Camilli. Synapsins in the Vertebrate Retina: Absence from Ribbon Synapses and Heterogeneous Distribution among Conventional Synapses. *Neuron* **5**, 19-33, doi:10.1016/0896-6273(90)90030-j (1990).
8. Maxeiner, S., F. Luo, A. Tan, F. Schmitz, T.C. Südhof. How to Make a Synaptic Ribbon: Ribeye Deletion Abolishes Ribbons in Retinal Synapses and Disrupts Neurotransmitter Release. *EMBO J* **35**, 1098-1114, doi:10.15252/embj.201592701 (2016).
9. Müller, T.M., K. Gierke, A. Joachimsthaler, H. Sticht, Z. Izsvak, F.K. Hamra, A. Fejtova, F. Ackermann, C.C. Garner, J. Kremers, J.H. Brandstätter, H. Regus-Leidig. A Multiple Piccolino-Ribeye Interaction Supports Plate-Shaped Synaptic Ribbons in Retinal Neurons. *J Neurosci* **39**, 2606-2619, doi:10.1523/JNEUROSCI.2038-18.2019 (2019).
10. Qiu, H., X. Wu, X. Ma, S. Li, Q. Cai, M. Ganzella, L. Ge, H. Zhang, M. Zhang. Short-Distance Vesicle Transport Via Phase Separation. *Cell* **187**, 2175-2193 e2121, doi:10.1016/j.cell.2024.03.003 (2024).
11. Regus-Leidig, H., M. Fuchs, M. Löhner, S.R. Leist, S. Leal-Ortiz, V.A. Chiodo, W.W. Hauswirth, C.C. Garner, J.H. Brandstätter. In Vivo Knockdown of Piccolino Disrupts Presynaptic Ribbon Morphology in Mouse Photoreceptor Synapses. *Front Cell Neurosci* **8**, 259, doi:10.3389/fncel.2014.00259 (2014).
12. Regus-Leidig, H., S. Tom Dieck, D. Specht, L. Meyer, J.H. Brandstatter. Early Steps in the Assembly of Photoreceptor Ribbon Synapses in the Mouse Retina: The Involvement of Precursor Spheres. *J Comp Neurol* **512**, 814-824, doi:10.1002/cne.21915 (2009).
13. tom Dieck, S., D. Specht, N. Strenzke, Y. Hida, V. Krishnamoorthy, K.F. Schmidt, E. Inoue, H. Ishizaki, M. Tanaka-Okamoto, J. Miyoshi, A. Hagiwara, J.H. Brandstatter, S. Lowel, T. Gollisch, T. Ohtsuka, T. Moser. Deletion of the Presynaptic Scaffold Cast Reduces Active Zone Size in Rod Photoreceptors and Impairs Visual Processing. *J Neurosci* **32**, 12192-12203, doi:10.1523/JNEUROSCI.0752-12.2012 (2012).

14. Usukura, J., E. Yamada. Ultrastructure of the Synaptic Ribbons in Photoreceptor Cells of Rana Catesbeiana Revealed by Freeze-Etching and Freeze-Substitution. *Cell Tissue Res* **247**, 483-488, doi:10.1007/BF00215740 (1987).

June 4, 2026

RE: JCB Manuscript #202509110R-A

Kaspar Gierke
Friedrich-Alexander-Universität Erlangen-Nürnberg

Dear Dr. Gierke:

Thank you for submitting your revised manuscript entitled "The missing link: Piccolino is essential for tethering synaptic vesicles to rod photoreceptor synaptic ribbons".

With this letter I am attaching the comments provided by reviewers who assessed your revised manuscript. Each of the three reviewers has a positive assessment of the revisions and experiments that you have made in response to their concerns regarding your original submission. The third reviewer assessing this revision and the second reviewer in his/her original review raised the possibility of a bridging protein(s). Considering the weakness and specificity of the binding you observed, I think that this should be considered in your discussion. With this concern addressed, your manuscript is one that the journal will be pleased to publish. Congratulations to you and your lab members.

To avoid unnecessary delays in the acceptance and publication of your paper, please read the following information carefully.

A. MANUSCRIPT ORGANIZATION AND FORMATTING:

Full guidelines are available on our Instructions for Authors page, http://jcb.rupress.org/submission-guidelines#revised.
**Submission of a paper that does not conform to JCB guidelines will delay the acceptance of your manuscript.**

1) Text limits: Character count for Articles is < 40,000, not including spaces. Count includes abstract, introduction, results, discussion, and acknowledgments. Count does not include title page, figure legends, materials and methods, references, tables, or supplemental legends.

2) Figures limits: Articles may have up to 10 main text figures.

3) Figure formatting: Scale bars must be present on all microscopy images, including inset magnifications. Molecular weight or nucleic acid size markers must be included on all gel electrophoresis. Aspect ratios of images may not be altered.

4) Statistical analysis: Error bars on graphic representations of numerical data must be clearly described in the figure legend. The number of independent data points (n) represented in a graph must be indicated in the legend. Statistical methods should be explained in full in the materials and methods. For figures presenting pooled data the statistical measure should be defined in the figure legends. Please also be sure to indicate the statistical tests used in each of your experiments (either in the figure legend itself or in a separate methods section) as well as the parameters of the test (for example, if you ran a t-test, please indicate if it was one- or two-sided, etc.). Also, if you used parametric tests, please indicate if the data distribution was tested for normality (and if so, how). If not, you must state something to the effect that "Data distribution was assumed to be normal but this was not formally tested."

5) Abstract and title: The abstract should be no longer than 160 words and should communicate the significance of the paper for a general audience. The title should be less than 100 characters including spaces. Make the title concise but accessible to a general readership.

6) Materials and methods: Should be comprehensive and not simply reference a previous publication for details on how an experiment was performed. Please provide full descriptions in the text for readers who may not have access to referenced manuscripts.

7) All antibodies, cell lines, animals, and tools used in the manuscript should be described in full, including accession numbers for materials available in a public repository such as the Resource Identification Portal. Please be sure to provide the sequences for all of your primers/oligos and RNAi constructs in the materials and methods. You must also indicate in the methods the source, species, and catalog numbers (where appropriate) for all of your antibodies. Please also indicate the acquisition and quantification methods for immunoblotting/western blots.

8) Microscope image acquisition: The following information must be provided about the acquisition and processing of images:
a. Make and model of microscope
b. Type, magnification, and numerical aperture of the objective lenses
c. Temperature

d. Imaging medium
e. Fluorochromes
f. Camera make and model
g. Acquisition software
h. Any software used for image processing subsequent to data acquisition. Please include details and types of operations involved (e.g., type of deconvolution, 3D reconstitutions, surface or volume rendering, gamma adjustments, etc.).

9) References: There is no limit to the number of references cited in a manuscript. References should be cited parenthetically in the text by author and year of publication. Abbreviate the names of journals according to PubMed.

10) Supplemental materials: There are strict limits on the allowable amount of supplemental data. Articles may have up to 5 supplemental figures. Please also note that tables, like figures, should be provided as individual, editable files. A summary of all supplemental material should appear at the end of the Materials and methods section.

11) eTOC summary: A ~40-50-word summary that describes the context and significance of the findings for a general readership should be included on the title page. The statement should be written in the present tense and refer to the work in the third person.

12) Conflict of interest statement: JCB requires inclusion of a statement in the acknowledgements regarding competing financial interests. If no competing financial interests exist, please include the following statement: "The authors declare no competing financial interests." If competing interests are declared, please follow your statement of these competing interests with the following statement: "The authors declare no further competing financial interests."

**13) ORCID IDs: ORCID IDs are unique identifiers allowing researchers to create a record of their various scholarly contributions in a single place. Please note that ORCID IDs are now *required* for all authors. At resubmission of your final files, please be sure to provide your ORCID ID and those of all co-authors.

14) A separate author contribution section following the Acknowledgments. All authors should be mentioned and designated by their full names. We encourage use of the CRediT nomenclature.

Please note that JCB now requires authors to submit Source Data used to generate figures containing gels and Western blots with all revised manuscripts. This Source Data consists of fully uncropped and unprocessed images for each gel/blot displayed in the main and supplemental figures. For assays performed using capillary electrophoresis and/or immunoassay-based detection, authors should instead provide the electropherogram graph(s) for each experiment, plotting fluorescence/chemiluminescence intensity vs. molecular weight/size. Please be sure to provide one Source Data file for each figure gels, blots, and/or capillary electrophoresis assays along with your revised manuscript files. File names for Source Data figures should be alphanumeric without any spaces or special characters (i.e., SourceDataF#, where F# refers to the associated main figure number or SourceDataFS# for those associated with Supplementary figures). For traditional gels and blots, the lanes of the gels/blots should be labeled as they are in the associated figure, the place where cropping was applied should be marked (with a box), and molecular weight/size standards should be labeled wherever possible. For capillary electrophoresis assays, each trace in the graph should be color-coded and labeled to indicate which protein, gene, or sample is being measured (please try to avoid red/green combinations to accommodate our color-blind readers).

Source Data files will be made available to reviewers during evaluation of revised manuscripts and, if your paper is eventually published in JCB, the files will be directly linked to specific figures in the published article.

Source Data Figures should be provided as individual PDF files (one file per figure). Authors should endeavor to retain a minimum resolution of 300 dpi or pixels per inch. Please review our instructions for export from Photoshop, Illustrator, and PowerPoint here: https://rupress.org/jcb/pages/submission-guidelines#revised

Journal of Cell Biology now requires a data availability statement for all research article submissions. These statements will be published in the article directly above the Acknowledgments. The statement should address all data underlying the research presented in the manuscript. Please visit the JCB instructions for authors for guidelines and examples of statements at (https://rupress.org/jcb/pages/editorial-policies#data-availability-statement).

B. FINAL FILES:

Please upload the following materials to our online submission system. These items are required prior to acceptance. If you have any questions, contact JCB's Managing Editor, Lindsey Hollander (lhollander@rockefeller.edu).

-- An editable version of the final text (.DOC or .DOCX) is needed for copyediting (no PDFs).

-- High-resolution figure and MP4 video files: See our detailed guidelines for preparing your production-ready images,

https://jcb.rupress.org/fig-vid-guidelines.

-- Cover images: If you have any striking images related to this story, we would be happy to consider them for inclusion on the journal cover. Submitted images may also be chosen for highlighting on the journal table of contents or JCB homepage carousel. Images should be uploaded as TIFF or EPS files and must be at least 300 dpi resolution.

**It is JCB policy that if requested, original data images must be made available to the editors. Failure to provide original images upon request will result in unavoidable delays in publication. Please ensure that you have access to all original data images prior to final submission.**

**The license to publish form must be signed before your manuscript can be sent to production. A link to the license to publish form will be sent to the corresponding author only. Please take a moment to check your funder requirements before choosing the appropriate license.**

Additionally, JCB encourages authors to submit a short video summary of their work. These videos are intended to convey the main messages of the study to a non-specialist, scientific audience. Think of them as an extended version of your abstract, or a short poster presentation. We encourage first authors to present the results to increase their visibility. The videos will be shared on social media to promote your work. For more detailed guidelines and tips on preparing your video, please visit https://rupress.org/jcb/pages/submission-guidelines#videoSummaries.

Thank you for your attention to these final processing requirements. Please revise and format the manuscript and upload materials within 14 days. If you need an extension for whatever reason, please let us know and we can work with you to determine a suitable revision period.

Please contact the journal office with any questions at cellbio@rockefeller.edu.

Thank you for this interesting contribution, we look forward to publishing your paper in Journal of Cell Biology.

Sincerely,

Louis Reichardt
Monitoring Editor
Journal of Cell Biology

Gabriele Stephan
Scientific Editor
Journal of Cell Biology

--------------------------------------------------------------------------------
Reviewer #1:

In their revised manuscript, Gierke et al. have adequately addressed my previous concerns with the following actions: 1) testing for expression of an N-terminal Piccolo fragment, 2) clarifying how SV tethering to the ribbon was measured, 3) explaining how Pclo cKO alters the shape of the synaptic ribbon in photoreceptors, and 4) changing the color scheme of the diagram in Figure 2. Based on these revisions, I support the publication of this study in Journal of Cell Biology. The EM images are beautiful and the data provide new information about the mechanism of synaptic vesicle tethering at ribbon synapses of photoreceptors.

Reviewer #2:

No further comments - the authors have adequately the reviewers' comments in my opinion

Reviewer #3:

The authors have largely addressed the comments raised by this reviewer. The revised manuscript is with improved quality. This reviewer will leave the editor to make the decision on whether a functional proof by re-expressing a Pclo mutant lacking the short ALPS motif identified to bind to SVs in the Pclo KO mice is required for the current manuscript. Another point is on the direct tethering of SV by the Piccolino ALPS-mediated binding to SV, both this reviewer and the reviewer #2 raised a possibility of a bridging protein(s), given the weak and non-specific binding between Piccolino ALPS and SV membranes. The authors probably should consider such possibility in the manuscript.
